# The impact of Disability Insurance reassessment on healthcare use

**DOI:** 10.1002/hec.4680

**Published:** 2023-04-01

**Authors:** Samia Badji, Anne Kavanagh, Dennis Petrie

**Affiliations:** ^1^ Centre for Health Economics Monash University Caulfield East Victoria Australia; ^2^ Melbourne School of Population and Global Health The University of Melbourne Melbourne Victoria Australia

**Keywords:** disability, healthcare use, reassessment, welfare benefits

## Abstract

Several Organisation for Economic Co‐operation and Development countries have constrained Disability Income Insurance (DI) eligibility and reassessed those on DI to encourage workforce participation. But these policies can also have unintended consequences. While receiving less income can directly worsen physical and mental health, the stress related to reassessment and the possibility of losing DI may also adversely affect mental health. This paper uses Australian population‐wide administrative data to explore how a 2014 policy ‐ where DI recipients under 35 were reassessed under stricter criteria ‐ affected healthcare use. We exploit this age targeting using a difference‐in‐difference regression design and find that the policy increased nervous system drug prescriptions (which includes antidepressants). Our findings suggest that the reassessment of DI recipients, even without income loss, may have had a significant negative impact on their mental health. DI reassessment policies may have the unintended consequence of worsening mental health and this needs be considered when deciding if reassessment is worthwhile.

## 1 INTRODUCTION

With the steep rise of Disability Insurance (DI) recipients, several Organisation for Economic Co‐operation and Development countries have tightened eligibility criteria and reassessed their current beneficiaries against stricter criteria (Low & Pistaferri, 2020). While these policies have decreased the number and cost of beneficiaries (Koning & Lindeboom, 2015; Parliamentary Budget Office, 2018), the unintended consequences of these reforms remain underexplored. The existing literature has largely focused on the impact on employment and hours worked of those removed from DI (Broadway et al., 2014; David et al., 2016; Deshpande, 2016). However, these policies may also have consequences outside of the labor market, in particular they may generate substantial stress^1^ resulting in worse (mental) health. These consequences need to be considered when deciding if reassessment is worthwhile. In the current paper, we estimate the impact on healthcare use of an Australian policy reform where DI recipients (also called Disability Support Pensioners) under the age of 35 were reassessed based on new eligibility criteria.

There are numerous mechanisms through which the reassessment process and outcomes of these reassessments may impact health. Those individuals removed from DI may be incentivized to work, and this work may have positive mental health impacts. But these individuals may be few and far between and it has been found that most of those removed from DI do not work (Bound, 1989; Chen & Van der Klaauw, 2008; French & Song, 2014; Jones, 2008; Moore, 2015; Von Wachter et al., 2011). Instead they often end up on unemployment benefits long‐term which in Australia and elsewhere provides significantly lower support than DI. Individuals on the margins of DI are likely still a vulnerable group and therefore reducing their income could put them at significant financial risk which could have flow on effects to their health. Lowering DI benefits has been shown to increase mortality (Gelber et al., 2018) and one can therefore fear that those removed who cannot find work are in a more precarious situation given their lower income. Those who do find work may also be putting their health at risk if working pressures worsen their medical conditions. Finally, it is possible that the threat of being reassessed or the reassessment process itself could negatively affect health, and in particular mental health, even for those who end up remaining on DI. These health effects may also impact healthcare use and related costs for the government and individual.

It is difficult to predict the overall impact of DI reassessment on healthcare use. On the one hand, the reassessment process itself may worsen health and increase healthcare need and in particular mental healthcare such as scripts for antidepressants. Also to remain on DI, individuals under reassessment had to prove that they still qualified under the new stricter criteria often based on expert reports, such as those from a General Practitioner (GP) or a Specialist. Individuals may therefore see their doctor more frequently than normal which may directly lead to increased use of medical services and potentially the number of medicines they are prescribed. On the other hand, for those removed from DI we may expect a negative impact on healthcare use through lower income or capacity to pay (or anticipated lower income) since DI schemes are more generous than unemployment benefits.

In this paper, we analyze the effects on healthcare use of a 2014 Australian policy which reassessed DI recipients against tighter criteria. Using unique large‐scale administrative data linking social security information with healthcare utilization before and after the reform, we ask whether those at threat of reassessment had greater healthcare use (GP and Specialist visits) and in particular higher prescriptions for nervous system drugs (which includes antidepressants) after the reform.

The targeted nature of the policy which focused on those on DI under 35 allows for an accurate estimation of the impact of the reassessment of DI beneficiaries. By comparing those just above and just under the age cut‐off, before and after the reform we can obtain a robust estimate of the intention‐to‐treat (ITT) effect. Using difference‐in‐differences, we find that those under 35 and initially on DI had relatively more prescriptions (0.13 more scripts on average per year) for the nervous system than their older counterparts after the policy was implemented. They also increased their GP visits and Specialist visits by 0.26 and 0.06 additional visits on average per year. This estimation is based on the 22,281 sample of 29–31 year olds and the increase in nervous system scripts amounts to as many as 6,000 additional prescriptions (in 2015 and 2016) while the additional number of medical visits (GP and Specialists combined) amounts to as many as 13,000. Together, these additional service and script use led to an extra 1.35 Million AUD (approximately 1 Million USD) healthcare spending in government subsidy and out of pocket expenditures.^2^ The effect is strongest for men who were also more likely to be on unemployment benefits after the reform. The effect on nervous system scripts is also larger for those who were already on nervous system scripts before the reform suggesting a worsening of their health condition.

Other studies have examined the effect of stricter policies using survey data (Curnock et al., 2016) or aggregated data (Barr et al., 2016). Curnock et al. (2016) find using UK data that those on DI who find work have better physical and mental health than those who remain on DI while those who moved to unemployment benefits have better mental health. Their findings, however, are based on survey data with small sample sizes and the outcomes are self‐reported. Barr et al. (2016) find that area level trends in suicides, self‐reported mental health problems and antidepressant prescribing rates are positively correlated with increases in reassessments. Another study which examined the mortality effects of a reform that re‐examined recipients under stricter eligibility criteria, and cut benefits (Garcia‐Gomez & Gielen, 2018) found higher mortality rates for women and lower mortality rates for men. Our paper complements this literature by using panel administrative data at the individual level to examine how the Australian reassessment reform impacted on healthcare use of those targeted by the reform. We complement our analysis with additional administrative data to more accurately estimate who was treated. We hypothesize that beyond the impact of being removed from DI, the actual reassessment process and threat of removal can be extremely stressful.

The remainder of the paper is divided into four sections. Section 2 provides essential background on the 2014 reform. Section 3 describes the data. Section 4 presents the methodology. We present the empirical findings in Section 5 and the discussion/conclusion are in the last section.

## 2 BACKGROUND

### 2.1 What does it mean to be on DI in Australia?

The Australian DI scheme grants DI to a person with one or more impairments if they are assessed as having at least 20 points on Impairment Tables.^3^ Further, the impairment must prevent the individual from working more than 15 h a week and also prevent them from undertaking a training activity that would equip them for work within the next 2 years. There is no partial disability payment, that is, DI is granted only to those meeting the minimum impairment rating regardless of how severely their impairment affects their ability to work beyond the 20‐point threshold. The level of income support from DI may differ from one person to another because payment is determined by the income and asset level of the recipient.

### 2.2 New 2012 impairment tables and DI reassessment policy

In January 2012, the Australian government introduced new Impairment Tables to assess the eligibility of DI applicants.^4^ An applicants' impairment was still required to be at least 20 points but the new impairment tables focused more on assessing the level of functional impact of the impairment rather than their medical diagnosis. Ultimately, it meant that fewer people became eligible for DI.

The revised Impairment Tables were applied not only to new applicants but also to any existing DI recipients selected for review from January 1, 2012. However reviews of existing applicants were usually only undertaken when circumstances had changed such as earnings, assets or medical information. Annually, the Australian government was reviewing (for medical or other reasons) approximately 10% of DI recipients to confirm their ongoing eligibility. Cancellation or payment reduction due to a review was relatively low at approximately 10% (Australian National Audit Office, 2016).

### 2.3 Our reform of interest—2014 targeted DI reassessment of those younger than 35 years old

In the fiscal year 2014–2015, a special Budget measure was implemented to undertake the medical review of 28,000 DI recipients under 35 years old who were granted DI between 2008 and 2011. Those selected for review had a reported work capacity of 8–15 h per week and were not granted DI on “manifest grounds”.^5^ The review was aimed to take place from July 2014 over a period of 18 months.^6^ By the end of 2015, over 22,800 reviews had been finalized. This led to 14% of cancellation of DI payment,^7^ 83% of which were due to the application of the revised Tables (Department of Social Services ‐ Australian Government, 2016). Many appealed their cancellation and the great majority of DI recipients remained on DI even after the reassessment despite the stricter Impairment Tables.

### 2.4 Reassessment process

Those reassessed were first notified and then received a Job Capacity Assessment. The Job Capacity Assessment is a comprehensive assessment of their current impairment level and work capacity and also identifies any barriers they face in finding and/or maintaining work with the idea of improving their work capacity. The recipient was allowed to provide evidence and the assessor also has access to information about current and past medical conditions and disabilities as well as prior participation and employment history. Assessors can liaize with treating doctors and other health professionals as needed. Although an extension was available, DI recipients initially only had 21 days to respond to the review requirements (Joint Committee of Public Accounts and Audit, 2017) which was short given wait times to see some specialists may have been months.

DI recipients deemed no longer eligible after the review had the right and a financial incentive to request an internal appeal given that their DI access was maintained until a decision on their appeal was reached. Over half of the cancellations were appealed.^8^ The internal review appeal is usually referred to a Subject Matter Expert who in turn refers the matter to the Authorized Review Officer (ARO) unless their decision is in the favor of the claimant. If the DI agency confirms the cancellation after the internal appeal, individuals can still appeal in Court.

Those found ineligible for DI under the new Impairment Tables continued to receive payments for a further 42 days but were then transferred to standard unemployment benefits and required to train or attempt to find employment (“mutual obligations”). Between July 2014 and September 2016, 2800 DI recipients were transferred from DI to unemployment benefits. Of those transferred, as at December 2016, 99% of them were still on unemployment benefits (Joint Committee of Public Accounts and Audit, 2017). Most, if not all, of those reassessed were removed from DI in the 2015 or 2016 calendar years.^9^ People with disability on unemployment benefits may face challenges in meeting the mutual obligation requirements of training or trying to find work (Henriques‐Gomes, 2020) although those assessed as having a partial capacity to work may have fewer mutual obligations. Unemployment benefits are however much lower than DI benefits which are approximately twice as high.

Given the threat of lower payments and greater requirements in terms of trying to find work, DI recipients could have been put at particular stress. Anecdotal evidence suggests that the review process was indeed stressful with an individual, whose payment had been canceled, reported to have suffered major depression as a result.^10^ Further evidence suggested that the 21‐day response timeframe was particularly stressful and that the reviews were not always well targeted as those with manifest grounds should have been excluded but were not always (Joint Committee of Public Accounts and Audit, 2017). Therefore, even for those whose payments were not canceled, the reform nevertheless may have engendered subsequent stress. The reminder of the paper estimates how the healthcare use of DI recipients was affected by this reform.

## 3 ADMINISTRATIVE DATASETS AND SUMMARY STATISTICS

### 3.1 Linked administrative data on welfare receipt and out‐of‐hospital healthcare use

We exploit unique panel administrative data on healthcare use in Australia. The 2016 Multi‐Agency Data Integration Project (MADIP) Basic Longitudinal Extract (BLE) 2011–2016 Cohorts contains yearly data from 2011 to 2016 for the 2016 Australian resident population. These data stem from key Australian government administrative datasets including the: Medicare Benefits Schedule (MBS); Pharmaceutical Benefits Scheme (PBS); and the Social Security and Related Information (SSRI) database. The dataset further provides demographic information such as age, gender, Indigenous status and location at the Statistical Area Level 1 (SA1).^11^ A more extensive description of the data can be found in Saxby et al., (2020).

The SSRI database indicates whether someone received a certain payment type at any time during each calendar year. Social welfare payments include DI, unemployment benefits, allowances for studying, parental leave, allowances for primary carer, and sickness allowance. We can therefore infer whether someone was on DI at any time during each calendar year from 2011 to 2016 and also whether they accessed other payments. Because the dataset only provides an indicator for any payments in a given year an individual transferred from DI to unemployment benefits in a particular calendar year will be recorded as receiving both at least sometime during that year even though they cannot be on both benefits concurrently.

The healthcare use data come from the PBS (medications) and MBS (health services) components. The PBS component provides information on the number of prescriptions filled in the calendar year for out‐of‐hospital medicines many of which are subsidized by the federal government.^12^ It is broken down by type of scripts, in particular, we focus on the number of scripts for the nervous system (where antidepressants make up for a third of total scripts for the nervous system^13^) as the reform may have had an impact on mental health. We also consider changes in the use of other medicines and in particular those related to physical health though we expect the reform had little, if any, immediate impacts on physical health. The MBS component provides information on the number of federally subsidized medical visits, tests and services used by an individual during the calendar year disaggregated by types of visits or services. Specifically, we have the annual number of visits to the GP and visits to a Specialist. It is important to note that both the annual scripts and medical visits are top coded at 30 to maintain confidentiality. If individuals obtain health services or scripts that are not accessed through the MBS or PBS (which cover the majority of drugs and services), this service use will not appear in our data.^14^

### 3.2 Sample description

**FIGURE 1:**
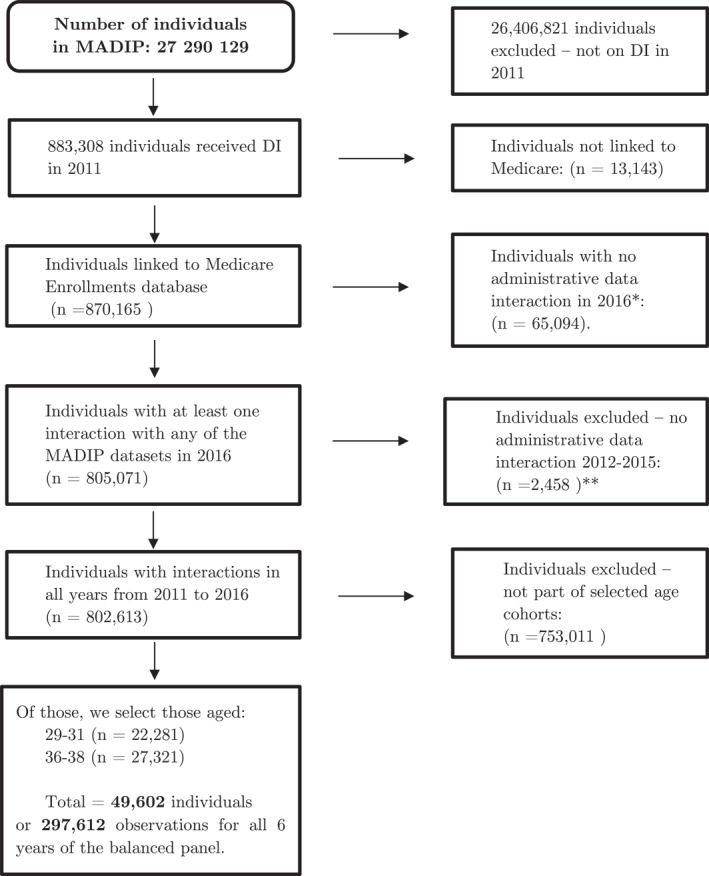
Flow chart of the sample selection for the main analysis. *Those with no interaction represent 7.5% of the total sample. This is mainly due to older age groups who may likely have passed away. If we first restrict to those aged 29–31 and 36–38, only 3.6% would have no interaction in 2016. An individual has an administrative interaction in 2016 with the Multi‐Agency Data Integration Project (MADIP) data if they either received some welfare benefits assistance, used at least one Medicare Benefits Schedule services or Pharmaceutical Benefits Scheme services, filled a tax declaration, have undertaken any training/studies accredited by the Department of Education and Training or responded to the 2016 census. ** For 2012–2015, the same condition applies for interaction with the MADIP dataset except that they may not have responded to the 2016 census.

We are interested in the change in healthcare use of DI recipients targeted by the reform, that is, those aged less than 35 years old (in 2014) and who entered DI between 2008 and 2011. We compare them to those aged more than 35 who also entered DI between 2008 and 2011. The earliest year in the dataset is 2011 and we therefore restrict our analysis to those on DI at some time during 2011.^15^ As we are interested in their healthcare use, we remove those who were not linked to the Medicare Enrollments Database. To avoid considering those who moved overseas or passed away as having zero healthcare use, we further restrict the sample to individuals for whom there were at least one interaction recorded per year with any of the MADIP datasets for all years from 2011 to 2016. For a given year, this means that an individual must have either received some welfare benefits assistance, used at least one MBS services or PBS services, filled a tax declaration, or have undertaken any training/studies accredited by the Department of Education and Training. For 2016, individuals are also included if they responded to the 2016 census. Figure 1 shows the number of observations retained at each step in the selection of the final sample.

Table 1 presents the characteristics of our sample for the cohort aged 29–31 years old in 2014 (the Young or treated group) in the first column and those aged 36–38 years old (the Old or control group) in the second column.^16^ The first part shows the average age of the two groups as at August 9, 2014^17^ and the gender composition. Within groups, there is not much skewness in the age distribution and those aged 29–31 are on average 30 years old and those aged 36–38 years old are on average 37 years old. Both samples are mainly comprised of men with 42.9% of the sample female for the younger group and 42.0% for the older group.

**TABLE 1:** Characteristics of DI recipients in the treated and control groups.

Mean (std deviation)	Treated group	Control group
	Younger	Older
	(29–31 y.o.)	(36–38 y.o.)
Demographics		
Age (yrs) as at August 9, 2014	30.02	37.03
	(0.817)	(0.820)
Female	42.9%	42.0%
	(49.5)	(49.4)
Received welfare support		
Disability Insurance 2011	100%	100%
	(0)	(0)
Disability Insurance 2016	91.5%	94.9%
	(28.0)	(21.9)
Unemployment benefits 2011	5.59%	5.70%
	(23.0)	(23.2)
Unemployment benefits 2016	4.13%	1.17%
	(19.9)	(10.8)
Health care use (use; any use in %)		
Nervous system scripts 2011	8.26; 61.6%	10.67; 70.6%
	(10.39) (48.6%)	(11.18) (45.6%)
Nervous system scripts 2016	9.50; 61.9%	11.71; 70.2%
	(11.20) (48.6%)	(11.74) (45.7%)
GP visits 2011	6.88; 87.1%	8.24; 89.6%
	(7.19) (33.5%)	(7.94) (30.5%)
GP visits 2016	7.65; 88.4%	8.77; 89.8%
	(7.64) (32.0%)	(8.17) (30.3%)
Specialist visits 2011	1.66; 33.1%	1.75; 34.8%
	(4.33) (47.1%)	(4.30) (47.6%)
Specialist visits 2016	1.72; 35.6%	1.74; 36.9%
	(4.23) (47.9%)	(4.19) (48.3%)
Observations	22,281	27,321

*Note*: Our sample consists of individuals linked to the Medicare Enrollments Database (MEDB) and aged 29–38 years old in 2014 who received at least one payment from DI in 2011 and who had any medical visit or medical service used, prescription, tax form filled, social security payment or who responded to the census in 2016. We excluded those who were 32, 33, 34 and 35 to match the sample used in the regressions. Number of visits and number of prescriptions are the total for a year. Receiving welfare support (Unemployment benefits or DI) means that the person received the welfare support at any point during the corresponding calendar year.

*Source*: MADIP Basic Longitudinal Extract 2016 data.

The second part of the table focuses on welfare support in 2011 and 2016 (the earliest and latest years available in our dataset). By construction, everyone in 2011 is on DI at least sometime so the probability to be on DI is 1 for both groups in 2011. There are still approximately 5% of our sample that were on unemployment benefits at some stage in 2011. This can be explained by individuals who either left DI and went on unemployment benefits in 2011 or entered DI after being on unemployment benefits in 2011. The proportion on unemployment benefits is roughly the same between our two groups in 2011. We now consider welfare recipiency after the reform. In 2016, 91.5% of our “treated” younger cohort still received DI at some point while this was 94.9% in the older “untreated” cohort. We also found that by 2016 more of the younger treated cohort had received unemployment benefits (4.13%) compared to the older untreated cohort (1.17%).

The third part of Table 1 investigates the use of healthcare for our two cohorts. The control group, comprised of individuals aged over 35, have, as expected given their older age, higher use of medical services and more nervous system scripts. By 2016, the number of nervous system scripts had increased for both groups, though slightly more in absolute terms for the younger treated cohort but was still higher for the older group. Turning to medical visits, it is clear that the number of GP visits increased, while the number of Specialist visits remained fairly stable from 2011 to 2016 for both the Young and Old cohorts.

### 3.3 Disability exit rates by age

**FIGURE 2:**
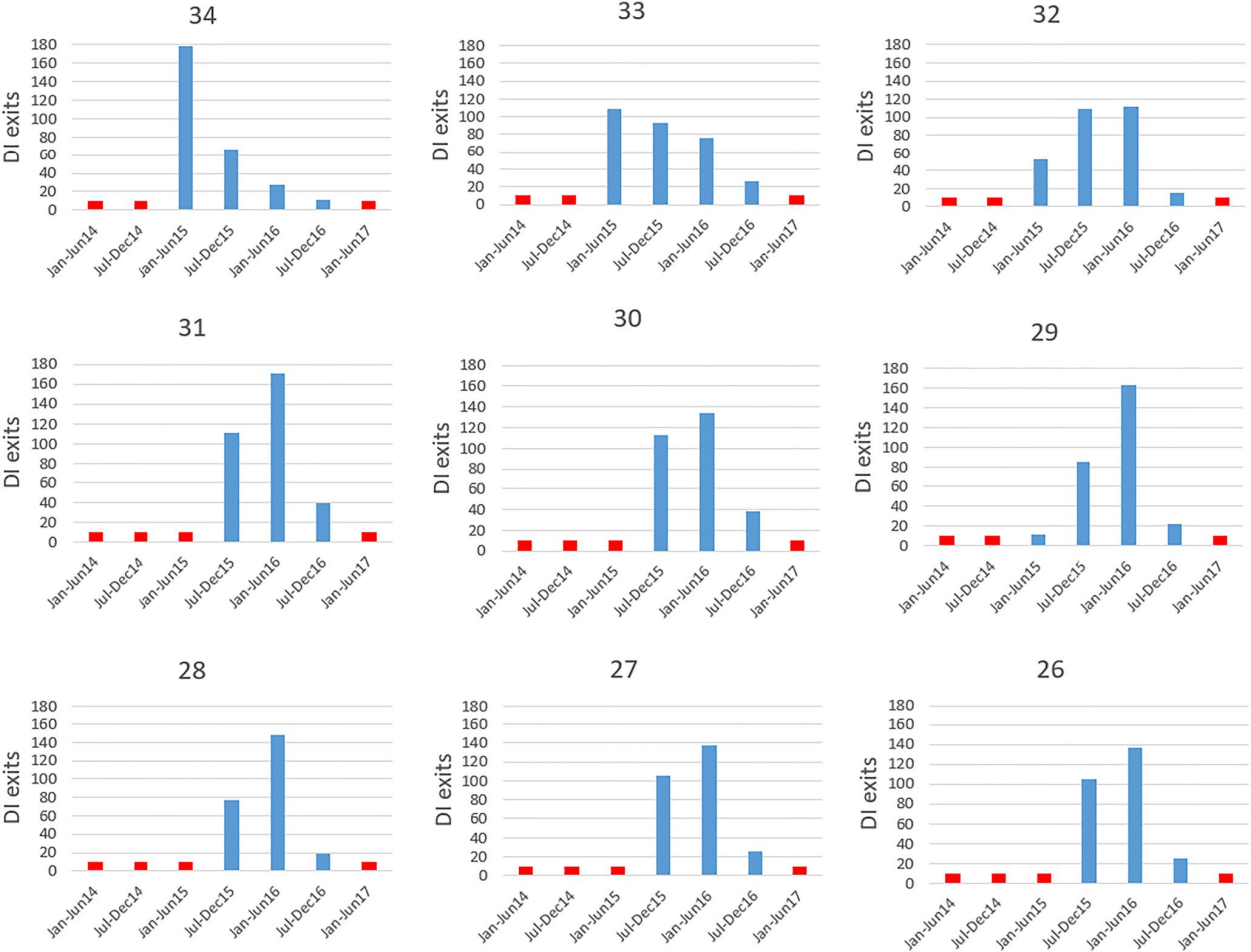
DI exits for each single age cohort for those aged 26–34 years old in 2014. Age is defined on July 01, 2014, date of the 2014 reform. Numbers below 10 bottom‐coded at 10 to ensure confidentiality and shown in red. The figures look similar whether exit numbers or exit rates are shown. *Source*: Authors' own calculation from the Australian Department of Social Services Data Over Multiple Individual Occurrences (DOMINO) dataset.

To understand how the reform took place, we rely on the Australian Department of Social Services Data Over Multiple Individual Occurrences (DOMINO) dataset which contains detailed event‐based data on individual welfare recipients throughout their interactions with the Department of Social Services from 2000 to 2016. We are interested in those who exited DI for “medical reasons” as those are likely due to our July 2014 reform of interest and the application of the new Impairment Tables. Figure 2 displays the number of removals from DI after January 2014 by 6‐month blocks for each 1‐year age cohort for those aged 26–34 years old in 2014 because their disability was assessed as short term or had impairment points below 20 (the two exit reasons likely to come from the 2014 reassessments).^18^

It is noteworthy that those aged 34 appear to be the first targeted group. They started heavily exiting DI as early as the first semester of 2015, most likely because they were about to turn 35 and therefore would have become ineligible for review. Very few exited DI in 2016. Those aged 33 and 32 years old appear to have been targeted next and exited as early as the first half of 2015. Only those aged 34 to 32 (the first row of graphs in Figure 2) seem to have experienced some early targeting while other age groups have relatively similar patterns. Those aged 31 and under all have relatively similar patterns and seem to have been mostly targeted after those aged 34 to 32 years old. For this cohort, there seems to be limited evidence of different timing of their reviews, at least as evidenced by their exit rates. They mainly started to exit DI in the second half of 2015 and more so in the first half of 2016. For all age groups, as of 2016, it seems like most reviews had been completed. None of those aged 35 and over seem to have been particularly targeted for those reasons consistent with the reform (see Figure A1 in the Appendix for DI exits for those aged 22–41 years old).

### 3.4 Welfare benefits recipiency over time

We examine how DI and unemployment benefits have evolved over the years 2011–2016 for those around the cut off, namely those aged 29–39 in 2014. We group together those aged 29–31 and those aged 36–38 as they are the two cohorts we focus on for our main analysis as discussed in Section 4.1.

**FIGURE 3:**
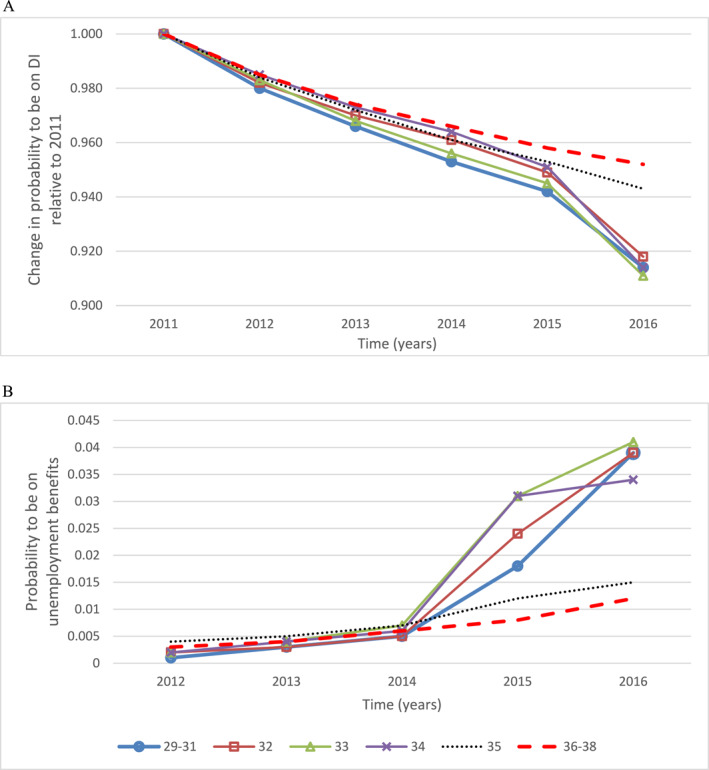
Probability to receive welfare benefits each year for different age cohorts. Panel (a) Disability Income Insurance (DI). Panel (b) Unemployment Benefits. Age cohorts are defined based on their age as of August 09, 2014. Our sample consists of individuals linked to the Medicare Enrollments Database (MEDB) and aged 29–38 years old in 2014 who received at least one payment from DI in 2011 and who, for each subsequent year had at least one medical visit or medical service used, prescription, tax form filled, social security payment or who responded to the census in 2016. We show those who were 32, 33, 34 and 35 years old separately as they are not part of our main sample. The data structure, with a simple binary indicator for receiving a certain welfare benefit at least at some time during the calendar year, implies that someone transitioning from DI to unemployment benefits in 2015 will appear as off DI only in 2016 while they will appear as on unemployment benefits as soon as 2015. This is why panel A is suggestive of some effect of the reform in 2016 while panel B shows some effect as early as 2015. In panel B, the year 2011 is omitted as it does not solely represent those who left DI but also those who transitioned onto DI from unemployment benefits. *Source*: MADIP Basic Longitudinal Extract 2016 data.

Figure 3 presents the probability to be on DI (panel A) and unemployment benefits (panel B) for at least 1 day during each calendar year. We focus on those who were on DI in 2011 as those not on DI in 2011 could not have been targeted by the policy. In panel A we can see that the probability to be on DI in 2011 is 1, consistent with our sample selection. For all age groups, the probability to be on DI is decreasing over time. It seems that the younger cohorts are slightly more likely to exit DI than the older cohorts, even before the reform. From the calendar years 2015–2016, one can see a significant drop in the probability to be on DI but only for those aged under 35 years old suggesting that the reform did take place with at least some individuals leaving DI in 2015 and never receiving DI at any time during 2016.

Panel B is similar to panel A but for the probability to be on unemployment benefits. As we restricted the sample to those on DI in 2011, Panel B helps understand the proportion of individuals who left DI and moved onto unemployment benefits. In 2011, the proportion on unemployment benefits is not only those who left DI but also those who were on unemployment benefits at the beginning of the calendar year and entered onto DI. For this reason, we do not present the number of people on unemployment benefits in 2011 as it can be misleading.

For all groups, the probability to be on unemployment is similarly increasing from 2012 to 2014. In 2015 and 2016, only those aged under 35 are much more likely to have entered unemployment benefits suggesting that the reform may have pushed people onto unemployment benefits. We see some effect as early as 2015 for unemployment benefits as people were transitioning from DI to unemployment benefits in 2015 and we see this increase further in 2016. Note that the graph for unemployment benefits is more helpful to understand the reform effects as it shows the likely year an individual left DI, although among those who left DI, a few may not have gone onto unemployment benefits but may have instead found work.

### 3.5 Prescription use over our time period

To illustrate how healthcare use evolved over the period for the treated and control groups from 2011 to 2016, we consider the change in use from 2011 for each group. We remove any age‐cohort‐specific effect by adding controls for each age‐cohorts and plot the coefficient associated with the difference from 2011 for each group‐year.

**FIGURE 4:**
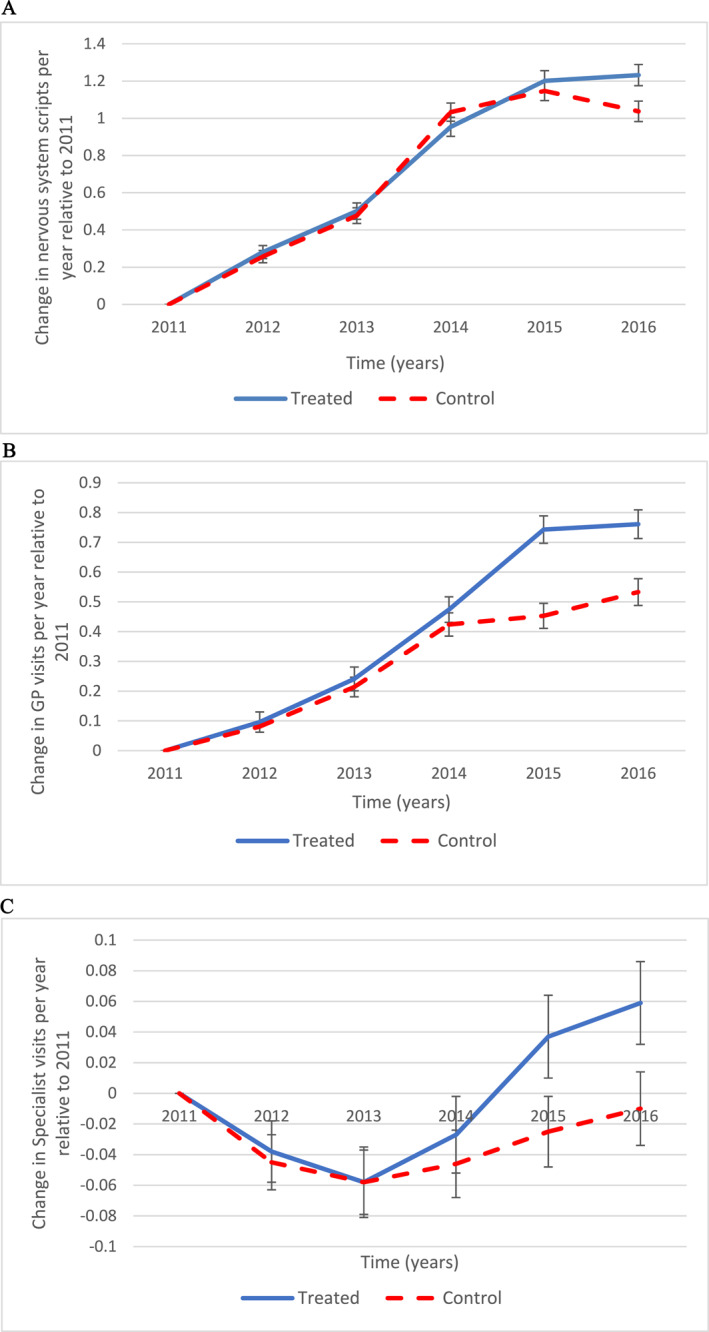
Absolute change in average healthcare use by year compared to 2011. Panel (a) Nervous system scripts before and after the July 2014 reform. Panel (b) GP visits before and after the July 2014 reform. Panel (c) Specialist visits before and after the July 2014 reform. Our sample consists of individuals aged 29–38 years old in 2014 who received at least one payment from DI in 2011 and who had any medical visit or medical service used, prescription, tax form filled, social security payment or who responded to the census in 2016. We excluded those who were 32, 33, 34 and 35. Each panel displays the coefficients associated with the interaction terms in the following equation:

We report the estimated average change in use for both groups over the years relative to 2011 in Figure 4. The trend for the *Young group* is represented by the blue line. The 2012 point is the estimated absolute difference in use between 2012 and 2011 and the last point represents the estimated absolute difference in use in 2016 compared to 2011 for the *Young group*. The red dashed line is similar to the blue line except that it reports those differences for the *Old group*.

$$H_{it}=\beta_{0}+\beta_{1}agecohort_{i}+\beta_{2}\;Young_{i}*\left(t=2012\right)+\text{…}+\beta_{6}\;Young_{i}*\left(t=2016\right)+\beta_{7}\;Old_{i}*\left(t=2012\right)+\text{…}+\beta_{11}\;Old_{i}*\left(t=2016\right)+u_{it}$$
Where $H_{it}$ *is* the healthcare use variable for individual *i* at year *t*. ${agecohort}_{i}$, are age‐cohort indicators (one for each year of age), *Young* is a dummy variable equal to 1 for the young (treated) group and 0 otherwise. *Old* is an indicator variable for whether individual *i* belongs to the old (control) group. Both the *Young* and *Old* indicator variables are interacted with year dummies. $u_{it}$ is an error of idiosyncratic terms. The coefficient $\beta_{2}$ represents the increase in the outcome from 2011 to 2012 for the young group. Similarly, if for example, the outcome is the number of nervous system scripts, $\beta_{11}$ would represent the increase in scripts for the nervous system from 2011 to 2016 for the old group. *Source*: MADIP Basic Longitudinal Extract 2016 data.

In Figure 4, each panel corresponds to a different regression with panel A displaying the trends for the number of nervous system prescriptions. The absolute growth in scripts for the Young (blue line) and Old (red dashed line) groups are very similar up until 2013. By 2016, the nervous system prescriptions have increased more for the young group and it seems the divergence started in 2015. Panel B corresponds to the number of GP visits. As can be seen, before 2014 the two lines were very close and following parallel trends. However, in 2014, the number of visits to the GP increased more for the young cohorts and the two lines start diverging. For Specialist visits (panel C), the differences in use compared to 2011 are very small. We see only very small differences between the young and old cohorts before 2014, but see that the young cohort starts diverging from the old cohort from 2014 onwards. Overall, for medical service use, both the number of visits to GP and Specialist have increased significantly more for the young cohorts after the 2014 reform relative to the old cohorts (and maybe even a slight increase in 2014).

Overall, Figure 4 is indicative of an increase in nervous system scripts for those targeted by the reform (compared to those untargeted), as well as increases in GP and Specialist visits. The next section presents the empirical specification to estimate the impact of the 2014 reform on healthcare use.

## 4 METHODOLOGY

### 4.1 Sample selection

As detailed in the background section, only those who entered DI between 2008 and 2011 were reassessed. Due to data limitations within MADIP, we cannot infer if someone who was on DI in 2011 had entered between 2008 and 2011. We also do not know who was reassessed. We therefore select all individuals who were on DI in 2011. We use the DOMINO dataset, which contains date of entry and exit from welfare payments, to estimate the proportion of individuals who were on DI in 2011 and under 35 in 2014 who actually entered DI between January 01, 2008 and December 31, 2011 and find that approximately 60% had entered DI in this timeframe.

For the following reasons we focus our analysis on those young “treated” individuals aged 29–31 years old on the August 9, 2014 and use those aged 36–38 years old in 2014 as the older “control” group (who were not in the targeted age range at any stage of the policy). Given that the reassessment was targeted at those under 35 at the time they received the notification of reassessment, it means that some DI recipients were on the borderline of being targeted. The reform potentially started to take place on the 1^st^ of July 2014 yet our data only provides age in years as of 9^th^ of August. That is, among those aged 35 on the August 9, 2014, some were already 35 on July 1, 2014 and could therefore not be targeted while others could be targeted for at most 6 weeks. Therefore, the youngest fully “untreated” cohort that can be selected for our control/old group were those aged 36 in 2014.

Although the oldest cohort who could have been “fully” targeted are those aged 34 at the time of the reform, there are several reasons why we select those aged 31 years old as the oldest cohort among our young/treated group. Someone who was 34 on the August 9, 2014 could only be targeted until they reach 35, that is, these individuals could not have been targeted in 2016 (but could have already received their reassessment notice and either been removed or appealed which could take approximately one year). Some of those aged 33 may also have no longer been targeted in 2016. On the other hand, someone who is 29 in 2014 could have been targeted for reassessment all the way until the end of 2016, the last year of our dataset. As it is not clear if those aged 33, 34 and 35 were targeted for all years after 2014, they are excluded from our sample.

It is noteworthy that among those aged under 33, the 32 years old have a distinct pattern of DI exit as described in Section 3.3. In order to have a homogenous treated group, we focus on those aged 29–31 for the young group, and take a similar 3‐year interval for the control/old group namely those 36–38 in 2014.^19^ Those aged 32, 33 and 34 years old are excluded because it is unclear whether they were targeted in all post reform years and they have a different treatment pattern, in particular their number of exits is higher in 2015 compared with 2016. Extending the age group to larger than a 3‐year interval means the treated and young group are more likely to differ and for this reason we chose to look at the 29–31 years old versus 36–38 years old. Table A4 panel B and panel C shows that similar results to our main analysis are also estimated for different age cohort choices.

### 4.2 Econometric model

We are interested in the 2014 policy which led to a reassessment for certain individuals under 35 years old on DI. Because the policy only targeted those under 35, it provides an optimal research design based on a difference‐in‐difference (DD) regression where the older “untargeted” cohort can be used as an appropriate control group:

(1) $$H_{it}=\beta_{0}+\beta_{1}T_{i}+\beta_{2}\;D_{t}+\beta_{3}\;T_{i}*D_{t}+\varepsilon_{it}$$

where $H_{it}$ is the healthcare use of individual *i* at time *t,* $T_{i}$ is a treatment group indicator equal to one if individual *i* is aged under 35 (Young cohort), and $D_{t}$ is a post reform indicator equal to one after the reform (for years 2015 and 2016^20^).

Given that our aim is to understand whether the reform disproportionately put those targeted by the reform (those <35 years old) under stress, our main healthcare variable H_it_ is nervous system scripts which includes antidepressants (but the same analysis is applied to our other outcomes of interest). $\beta_{1}$ captures the difference in scripts prior to the reform for those in the treated or targeted group compared to the untreated older cohorts while $\beta_{2}$ captures how the script use changes from the pre to post reform period for the older cohort. $\beta_{3}$ our coefficient of interest captures any differences in the absolute change in script use pre to post reform for those in the “treated” younger cohort compared to the change observed in the older untreated cohort. As there were no other specific reforms between 2014 and 2016 that could have influenced healthcare use for those under 35 differently from those over 35, we expect $\beta_{3}$ to reflect the effect of the 2014 policy reassessment.^21^ It should be noted that the coefficient $\beta_{3}$ reflects the average effect across all the younger cohort even though some may fall outside the particular targeted group (e.g., due to having a “manifest” condition), others may never have been targeted, some were reassessed but found to still be eligible, others were found ineligible but successfully appealed while others were ineligible and were removed from DI.

Equation (1) has minimal controls but we estimate alternative specifications that vary by the inclusion of 1‐year age cohort fixed effects, local area fixed effects and individual fixed effects to explore the robustness of the conclusions. The inclusion of 1‐year age cohort fixed effects captures any age related patterns associated with healthcare use. Local area fixed effects help account for differences across local areas that do not vary over time. Finally, individual fixed effects will capture differences that are individual specific and time invariant and will therefore also capture cohort effects. We expect the effect of the reform to be similar across these specifications.^22^

We also estimate a more flexible equation that estimates the effect for each treatment year (including 2014):

(2) $$H_{it}=\beta_{0}+\beta_{1}T_{i}+{\beta_{2}\;{Year2014}_{t}+\beta_{3}\;T_{i}*{Year2014}_{t}+\beta }_{4}\;{Year2015}_{t}+\beta_{5}\;T_{i}*{Year2015}_{t}+\beta_{6}\;{Year2016}_{t}+\beta_{7}\;T_{i}*{Year2016}_{t}{\;+\;\varepsilon }_{it}$$

In this specification, $\beta_{3}$ ($\beta_{5}$ and $\beta_{7}$ respectively) captures differences in the outcome in 2014 (2015 and 2016 respectively) relative to the pre reform years (2011, 2012 and 2013) for those in the “treated” younger cohort compared to the change observed in the older untreated cohort. Note that we include 2014 and the interaction between 2014 and the treatment group to understand whether there was any observable effect in 2014 (given the reform started to be implemented in late 2014). We expect $\beta_{3}$ to not be significantly different from zero. Using this specification on subsamples of people aged under 35 also helps us shed light on the mechanisms at stake and better understand the dynamics of the treatment effect.

### 4.3 Parallel trend assumption

The DD regression and the interpretation of $\beta_{3}$ holds under the assumption that the trends in healthcare use between the two groups would have remained parallel after the reform had the reform not happened. Figure 3 can help understand whether this is likely to hold. If the trends were parallel before the reform, it provides some confidence that these trends would have stayed parallel after the reform, had the reform not taken place. As described earlier, the younger and older cohorts do not seem to have significantly different trends before the reform. This assumption will also be tested by including an interaction term between our treatment group and the trend before 2014 *pretrend*. A statistically significant $\beta_{5}$ in Equation (2) below would be indicative of evidence of non‐parallel trends before the reform. We report the *p*‐value for $\beta_{5}$, where a *p*‐value below 0.05 would indicate evidence that the DD analysis may be producing bias results.

(3) $$H_{it}=\beta_{0}+\beta_{1}T_{i}+\beta_{2}\;D_{t}+\beta_{3}\;T_{i}*D_{t}+\beta_{4}\;{pretrend}_{t}+\beta_{5}\;T_{i}*{pretrend}_{t}+\varepsilon_{it}$$

When *p*‐values suggest evidence of biased results due to differing trends, it is possible to provide an alternative estimate. When the *p*‐value is below 0.05, we report the value of the estimated coefficient for $\beta_{5}.$ ^23^ We then provide an alternative estimate for the interaction term $\beta_{3}$ in Equation (1) which simply subtracts 3*${\hat{\beta }}_{5}$ in Equation (3)^24^ to $\hat{\beta_{3}}$ in Equation (1), that is we provide a value for $\hat{\beta_{3}}$ which accounts to the non parallel pre reform trends by assuming that they would have kept their pre reform linear divergence after the reform had the reform not happened.

We also run placebo analyses and test whether there is any interaction effect between the young cohort and alternative post reform indicators, namely post 2011, post 2012 and post 2013. This would also tell us whether there were major changes between the two groups prior to the reform which may bias our estimate. It is worth noting that we may expect some effect in 2014 given the reform started in July 2014 though the reform likely took some time to get started at least for some age groups.

### 4.4 Other prescriptions

If nervous system scripts increase as well as visits to the GP and the Specialist, it could be that every types of script increased. We therefore explore all other scripts. We hypothesize that other scripts are less likely to change as a result of stress. If we assume that there is no particular reason for identifying, say, dermatology issues more than identifying nervous system issues as a result of the increase in GP and Specialist visits, then having on the one hand no significant effect for those scripts and on the other hand an increase in scripts for nervous system would strengthen the hypothesis that those who were reassessed were under significant stress. We therefore examine other scripts to shed light on the mechanisms at stake.

As there are 15 types of scripts in total, testing each script independently can be misleading. If the reform had no effect on any of the scripts, the probability to find that there is at least one script with an interaction term significantly different from zero at 5% is about 50% (1–0.95^15). To reduce the likelihood of assuming that there is an effect of the reform when there is actually none, we adopt Anderson (2008)'s sharpened *q*‐values approach which accounts for multiple hypothesis testing.

### 4.5 Financial implications of the reform on healthcare costs

Not only do we examine the potential stress impact induced by the reform but we also consider the additional healthcare costs which were induced by the reform. We use the results from the DD regression to understand which types of visits and scripts are affected by the reform. Where absolute changes in healthcare use are significant (i.e., where $\beta_{3}$ in Equation (1) is significant), we multiply the additional healthcare use (visits or scripts) by the average cost for a visit or a script in 2016 (see Table A3) to obtain the average additional cost per individual per year. We then multiply by the number of individuals in our treated group and the number of treated years. The MADIP BLE data contain both out of pocket costs and government subsidies so that both the cost to the individual and the cost to the government can be considered. We use publicly available information^25^ on how much DI and unemployment recipients were receiving in July 2016 to examine the financial gains in terms of welfare benefits from the government perspective.

## 5 RESULTS

### 5.1 Main results

Table 2 presents the results of Equation (1) (with minimal controls), the difference‐in‐difference specification on the healthcare use outcomes. The first column presents the results for the number of scripts for the nervous system (which include antidepressants), the second and third columns the number of GP and Specialist visits respectively. The estimated Young*Post2014 coefficient represents the absolute additional change in healthcare use after the reform for those in the younger treated group relative to the change experienced by the control group. The results indicate that the reform led to an average 0.13 (0.013 of a standard deviation) per year increase in the number of scripts for the nervous system for each individual in the “treatment” cohort. In terms of extensive margin, Table A6 in the Appendix shows that an additional 6 in 1000 targeted individuals were filling scripts for the nervous system each year after the reform.

**TABLE 2:** Difference‐in‐difference regression on healthcare use outcomes and welfare recipiency.

	Scripts	Medical visits	
	Nervous system	GP	Specialist
Young*Post2014	0.132**	0.236***	0.059**
	(0.053)	(0.044)	(0.024)
Young (“treated”)	−2.414***	−1.325***	−0.081**
	(0.093)	(0.061)	(0.033)
Post 2014	0.650***	0.313***	0.020
	(0.036)	(0.031)	(0.016)
Constant	11.112***	8.416***	1.714***
	(0.065)	(0.043)	(0.022)
Observations	297,612	297,612	297,612
Pretrend diff. (*p*‐val)	0.312	0.424	0.657

*Note*: Standard errors in parentheses clustered at the individual level. The last row indicates the probability that the trends were similar for the old and the young groups prior to 2014 (it tests whether the trend before 2014 for the young group was statistically significant conditional on the general trend before 2014 and the covariates of the regression). The group of young includes those aged 29–31 years old in 2014. The group of old (control group) includes individuals aged 36–38 years old in 2014.

*p < 0.1, **p < 0.05, ***p < 0.01.

*Source*: MADIP Basic Longitudinal Extract 2016 data.

Such changes in scripts are unlikely to occur without a visit to the GP or the Specialist. The results for medical visits suggest that the reform increased GP visits by 0.24 (0.036 of a standard deviation) per year per individual and Specialist visits by 0.06 (0.014 of a standard deviation) per year per individual. Results for the extensive margin in Table A6 suggest that 10 per 1000 more targeted individuals saw their GP and 6 per 1000 more saw a Specialist each year after the reform. As explained in the background section, to remain on DI, medical evidence was needed so that some individuals at threat of removal likely went to the GP to gather medical evidence to contest their potential removal from DI. Therefore, the increase use of GP and Specialist services are likely to be a combination of visits to remain on DI and potentially visits that could be due to stress induced as part of the review process. For all our healthcare use outcomes, the hypothesis that the trends were parallel (*p*‐value in the last row) cannot be rejected at the 5% level.

To better understand the scope of the reform and the unintended costs of the reform, one can estimate how many additional scripts and visits were made due to the reform. For those aged 29–31 years old in 2014, that is 22,281 individuals, there is on average a 0.132 increase in the number of scripts per year after 2014. This amounts to an extra 6000 scripts for nervous system due to the reform. For the medical visits, this amounts to 10,500 additional GP visits and 2600 additional Specialist visits.

### 5.2 Alternative specifications

Table 3 presents the results of alternative specifications for the DD analysis for our main outcome of interest, the annual nervous system scripts. The specifications vary due to the inclusion of 1‐year age cohort fixed effects, local area fixed effects and individual fixed effects as indicated in the last rows for each column. The first column repeats the specification used in Table 2 for ease of comparison.

**TABLE 3:** Alternative specifications for the difference‐in‐difference regression on the number of prescriptions for nervous system.

	(1)	(2)	(3)	(4)	(5)
Young*Post2014	0.132**	0.132	0.124**	0.124**	0.132**
	(0.053)	(0.088)	(0.053)	(0.053)	(0.053)
Young (“treated”)	−2.414***			−2.253***	
	(0.093)			(0.136)	
Post 2014	0.650***	0.650***	0.667***	0.667***	0.650***
	(0.036)	(0.059)	(0.038)	(0.036)	(0.036)
Constant	11.112***	11.190***	11.204***	11.059***	10.028***
	(0.065)	(0.051)	(0.092)	(0.062)	(0.009)
Fixed effects	No	No	SA2	SA1	Indiv
Cohort dummies	No	Yes	Yes	No	N/A
Clustered std error	Yes	No	No	No	Yes
Observations	297,612	297,612	296,070	296,070	297,612

*Note*: Standard errors in parentheses. Clustered standard errors are clustered at the individual level. The last row indicates the probability that the trends were similar for the old and the young prior to 2014 (it tests whether the trend before 2014 for the young group was statistically significant conditional on the general trend before 2014 and the covariates of the regression). The group of young includes those aged 29–31 years old in 2014. The group of old (control) includes individuals aged 36–38 years old in 2014.

*p < 0.1, **p < 0.05, ***p < 0.01.

*Source*: MADIP Basic Longitudinal Extract 2016 data.

The table shows that our conclusions are robust to alternative specifications. Column 2 shows the results with age‐specific cohort dummies instead of the young and old dichotomy. This specification is equivalent to using age fixed effects. In line with the main results, on average the number of scripts for the nervous system increases by 0.13 per year per individual. The third column adds control for where individuals live at the Statistical Area Level 2 (SA2)^26^ and suggests a similar 0.12 increase in prescriptions. If instead SA1s are used, which are smaller areas nested within an SA2, and if one uses the young dummy instead of the cohort controls, the results remain very similar. The last column uses individual fixed effects and the results are again similar. Comparing the first column with column (5) allows to understand how time invariant individual characteristics can affect the results and again we find very comparable results.^27^

### 5.3 Heterogeneity

The reform did not impact everyone in the same way. Table 4 presents the result of the regression disaggregated by gender. Although in general the number of nervous system scripts are higher for women (as evidenced by the coefficient on the constant), men experienced a greater increase in nervous system scripts due to the reform. Although this gender difference was not statistically significant, the higher coefficient would be in line with a higher stress given that men were also more likely to be on unemployment benefits after the reform. This contrasts with similar policies in the Netherlands (Garcia‐Gomez & Gielen, 2018) which found a greater increase in mortality rates for women than for men. However in the Netherlands case, reassessments very often led to income loss whereas in our situation, most individuals reviewed remained on DI.

**TABLE 4:** Difference‐in‐difference regression on healthcare use outcomes, by gender.

	Panel A: Women			Panel B: Men		
	Scripts	Medical visits		Scripts	Medical visits	
	Nervous system	GP	Specialist	Nervous system	GP	Specialist
Young*Post2014	0.114	0.123*	0.072*	0.147**	0.319***	0.050*
	(0.081)	(0.072)	(0.042)	(0.069)	(0.056)	(0.027)
Young (“treated”)	−2.464***	−0.910***	−0.022	−2.391***	−1.676***	−0.139***
	(0.145)	(0.097)	(0.061)	(0.121)	(0.075)	(0.036)
Post 2014	0.583***	0.370***	0.009	0.699***	0.272***	0.027
	(0.054)	(0.049)	(0.028)	(0.049)	(0.040)	(0.018)
Constant	11.681***	9.904***	2.230***	10.699***	7.336***	1.339***
	(0.102)	(0.067)	(0.040)	(0.084)	(0.055)	(0.025)
Observations	126,270	126,270	126,270	171,342	171,342	171,342
Pretrend diff. (*p*‐val)	0.170	0.493	0.626	0.901	0.654	0.908

*Note*: Standard errors in parentheses clustered at the individual level. The *p*‐value in Pretrend diff. indicates whether there the pretrend differed between the old and the young prior to 2014 (it gives the probability that the young pretrend was statistically significant conditional on the general pretrend and the covariates of the regression).

*p < 0.1, **p < 0.05, ***p < 0.01.

*Source*: MADIP Basic Longitudinal Extract 2016 data.

We also look into the effect for those who may have been more mentally vulnerable by focusing on those already on nervous system scripts before the reform. Table 5 shows that the effects on nervous system scripts are stronger for these people despite having similar effects on GP and Specialist visits. Given that most drugs can come in higher dosage, this suggests that they started filling scripts more frequently or increased the different types of nervous system medicines. This would be consistent with the explanation that the reform led to increased stress.

**TABLE 5:** Difference‐in‐difference regression on healthcare use outcomes for those who already had scripts for the nervous system.

	Scripts	Medical visits	
	Nervous system	GP	Specialist
Young*Post2014	0.202***	0.227***	0.059*
	(0.073)	(0.060)	(0.034)
Young (“treated”)	−1.702***	−1.087***	0.128***
	(0.111)	(0.079)	(0.048)
Post 2014	0.277***	0.147***	−0.037*
	(0.047)	(0.040)	(0.021)
Constant	15.261***	9.988***	2.099***
	(0.072)	(0.053)	(0.030)
Observations	198,114	198,114	198,114
Pretrend diff. (*p*‐val)	0.865	0.814	0.319

*Note*: Standard errors in parentheses clustered at the individual level. The last row indicates the probability that the trends were similar for the old and the young groups prior to 2014 (it tests whether the trend before 2014 for the young group was statistically significant conditional on the general trend before 2014 and the covariates of the regression). The group of young includes those aged 29–31 years old in 2014. The group of old (control group) includes individuals aged 36–38 years old in 2014. Both groups are restricted to those who had at least one script for nervous system in 2011.

*p < 0.1, **p < 0.05, ***p < 0.01.

*Source*: MADIP Basic Longitudinal Extract 2016 data.

Table 6 shows the heterogeneity of the treatment effect of the reform for different treatment years (as displayed in Equation (2)) for our main treated group (those aged 29–31 in 2014) and those aged 32–34 in 2014 who seemed to have been the first targeted as described in Section 3.3. We use a consistent control group to compare the results across the treated age cohorts. Although we consider the year 2014 as a pre treatment year in our main specification, we show the estimated effect in 2014 because those aged 34 in 2014 were potentially already being targeted with reviews toward the end of 2014 given that many had already exited DI in the first half of 2015.

**TABLE 6:** Difference in difference regressions with heterogeneity in post treatment effect for years 2014–2016 for different age cohorts.

Interaction term		Scripts	Medical visits	
		Nervous system	GP	Specialist
**29–31** versus 36–38 (main specification)	Young*2014	−0.096*	0.036	0.016
		(0.053)	(0.047)	(0.026)
	Young*2015	0.037	0.276***	0.060**
		(0.062)	(0.053)	(0.029)
	Young*2016	0.179***	0.214***	0.067**
		(0.068)	(0.057)	(0.031)
**34** versus 36–38	Young*2014	0.188**	0.071	0.023
		(0.078)	(0.064)	(0.035)
	Young*2015	0.264***	0.044	0.005
		(0.089)	(0.074)	(0.039)
	Young*2016	0.228**	0.009	−0.053
		(0.097)	(0.080)	(0.041)
**33** versus 36–38	Young*2014	−0.014	0.124*	−0.007
		(0.075)	(0.066)	(0.036)
	Young*2015	0.055	0.104	0.001
		(0.087)	(0.074)	(0.040)
	Young*2016	0.116	0.092	−0.011
		(0.095)	(0.081)	(0.044)
**32** versus 36–38	Young*2014	0.080	0.080	0.080**
		(0.076)	(0.065)	(0.036)
	Young*2015	0.226***	0.277***	0.128***
		(0.088)	(0.073)	(0.041)
	Young*2016	0.274***	0.183**	0.120***
		(0.097)	(0.079)	(0.045)
**32–34** versus 36–38	Young*2014	0.117**	0.086*	0.041
		(0.055)	(0.047)	(0.025)
	Young*2015	0.206***	0.134**	0.046*
		(0.063)	(0.053)	(0.028)
	Young*2016	0.258***	0.103*	0.014
		(0.069)	(0.057)	(0.030)

*Note*: Standard errors in parentheses clustered at the individual level. The regression results correspond to Equation (2). No significant pretrend was detected at the 10% level for any of those regressions. Full table of regressions available upon request.

*p < 0.1, **p < 0.05, ***p < 0.01.

*Source*: MADIP Basic Longitudinal Extract 2016 data.

For those aged 29–31 years old (our key treatment group), we do not see a significant increase in nervous system scripts in 2014. There seems to be a small negative effect in 2014 although it is only significant at 10%. The effect in 2016 is significant and about five times larger than the corresponding effect for 2015 which is by itself not statistically significant. The increase in GP and Specialist visits seems relatively similar for 2015 and 2016. Overall, the results suggest that directly following the reform there was an increase in GP and Specialist visits, most likely due to individuals gathering evidence to justify remaining on DI. The subsequent increase in nervous system scripts could be explained by the stressful process of the review.

We now focus on those reviewed first (those aged 32–34 in 2014.) Those aged 34 in 2014 have higher nervous system scripts as early as 2014 compared to their use in 2011–2013. This is the only age group for which we see a positive and significant effect on scripts in 2014. This is also the only age group in Figure 2 with higher DI exits in the first half of 2015 compared to all other 6‐month blocks suggesting that this age group was targeted very early on after the reform started. It is possible that this age group was put under significant stress as early as the second half of 2014. They were likely targeted early because, people had to be under 35 at the time the review notice was sent and thus for many in this age cohort it was likely now or never to be reviewed. It is noteworthy that for nervous system scripts, the estimated reform effect in 2016 is still quite large. This suggests that the healthcare use associated with the stress of the reassessment may have long lasting effects although it could also be due to some people appealing and still having their case under consideration in 2016. It is also important to highlight that the use of antidepressants is often persistent and therefore if some individuals were stressed, even for a short amount of time, they may have still been put on antidepressants for a longer amount of time given the withdrawal effects and unclear guidelines for cessation (Kendricks, 2021). These effects could diminish after 2016 but our current data cannot capture this. It is also noteworthy that the scripts for nervous system had already increased as early as 2014 for this age group, even though they were only removed from DI in 2015 (Figure 3 panel B shows that the probability that they were on unemployment benefits only started to increase in 2015 and Figure 2 shows that DI exits only started in the first half of 2015). This suggests that although the loss of income may play a role in generating stress, the reassessment and potentially the stress of losing income in itself likely generated stress prior to the potential DI removal. GP and Specialist visits of those aged 34 do not seem to have increased directly after the reform relative to the control group. It could be that they did not have the time to see their GP or Specialist to gather the medical evidence required given the quick targeting of this age group.

For those aged 33 in 2014, we see a general increase of their nervous system scripts although the estimated reform effects in 2015 and 2016 are not significant. Those aged 33 in 2014 seem to have started seeing their GP as early as 2014 and the magnitude is similar in 2015 and 2016 although none of the estimated reform effects are significant at 5%. There does not seem to be an increase in Specialist visits which could be due to the rapid targeting of those aged 33 and the generally longer waiting time to see a Specialist. Therefore, it could be that those aged 33 had the time to see a GP but not a Specialist. For those aged 32, the increase in nervous system scripts is large in particular for the years 2015 and 2016. They seem to have increased their GP and Specialist visits especially in 2015 and 2016 which matches what we would expect given they were targeted later.

### 5.4 Mechanisms

We explore the extent to which the policy increased scripts in general or whether this increase was concentrated on scripts related to stress (i.e., nervous system scripts). Table 7 presents the estimated coefficient for the interaction term of the DD regression for each type of scripts.^28^ Out of the 15 script types, five showed a significant policy effect at the 5% level namely those for the nervous system, the musculoskeletal system, the alimentary system, cardiovascular scripts and anti‐infectives scripts. For the latter three the estimated effect is negative, however, the parallel trend assumption appears violated with differing pre‐reform trends among the treated and control group. Assuming the pre reform trends would have kept their linear trajectory after 2014, the effects become very close to zero (see line “Reform effect with correction for pre trends”). Therefore, only the scripts for the nervous system and the musculoskeletal system show a positive and significant effect at 5%. Given we have tested several types of scripts, and thus increased the chance of finding a significant result, in addition to the *p*‐values shown in brackets, we also show the sharpened *q* value in curly brackets. Accounting for false discovery rates, we find that only the interaction term related to scripts for the nervous system is significant at 5%.

**TABLE 7:** Annual number of prescriptions for the nervous system and other types of prescriptions.

	Nervous system	Musculo‐skeletal system	Alimentary system	Cardiovascular	Anti‐infectives	Respiratory system	Blood, blood forming organs	Genitourinary, sex hormones
Young*Post2014	0.132**	0.026**	*−0.124*^^^	*−0.214*^^^	*−0.046^^*	−0.027	−0.013	0.000
	(0.053)	(0.013)	(0.028)	(0.025)	(0.020)	(0.017)	(0.010)	(0.009)
	[0.012]	[0.046]	[0.000]	[0.000]	[0.022]	[0.104]	[0.167]	[0.992]
	{0.055}	{0.113}	{0.001}	{0.001}	{0.071}	{0.210}	{0.314}	{1.000}
Observations	297,612	297,612	297,612	297,612	297,612	297,612	297,612	297,612
Pretrend diff. (p‐val)	0.312	0.456	0.000	0.000	0.004	0.151	0.035	0.398
Reform effect with correction for pre trends:			0.020	0.047	0.038		0.014	

	Sensory organs	Dermatology	Systemic hormonal	Antineoplastic and immunomodulating agents	Antiparasitic, insecticides	Various scripts	Other scripts
Young*Post2014	−0.002	0.005	0.004	0.005	−0.000	−0.002	−0.000
	(0.010)	(0.007)	(0.008)	(0.011)	(0.001)	(0.004)	(0.002)
	[0.831]	[0.489]	[0.635]	[0.658]	[0.880]	[0.584]	[0.861]
	{1.000}	{0.918}	{0.918}	{0.918}	{1.000}	{0.918}	{1.000}
Observations	297,612	297,612	297,612	297,612	297,612	297,612	297,612
Pretrend diff. (p‐val)	0.986	0.532	0.130	0.190	0.572	0.001	0.860
Reform effect with correction for pre trends:						0.013	

*Note*: Cells in italics are those for which there was a pretrend detected at least at the 10% level. Standard errors in parentheses clustered at the individual level. *p*‐values in squared brackets. Sharpened False Discovery Rate (FDR) *q*‐values shown in curly brackets. The last row indicates the probability that the trends were similar for the old and the young groups prior to 2014 (it tests whether the trend before 2014 for the young group was statistically significant conditional on the general trend before 2014 and the covariates of the regression). For *p* values below 0.1, we show the coefficient for the interaction term corrected for the young specific trends before the reform, that is we subtract 3*the coefficient on the young specific pre trend in the regression of Equation (3) shown in table A11. The group of young includes those aged 29–31 years old in 2014. The group of old (control group) includes individuals aged 36–38 years old in 2014.

*p < 0.1, **p < 0.05, ***p < 0.01; *p* < 0.01; ^*p* < 0.1, ^^*p* < 0.05, ^^^*p* < 0.01 for cells in italics.

*Source*: MADIP Basic Longitudinal Extract 2016 data.

While our results are indicative of stress induced by the reform, it is difficult to infer exactly which part of the reform may have led to increased stress. We conjecture that the process of medical reassessments with the potential to be on lower income was particularly stressful and that our results are unlikely to be solely driven by those who actually lost their benefits. This is because the reform led to very few removals as detailed in Section 2.3 and most people in our sample were still on DI in 2016 as shown in Table 1. This conjecture is also supported by Figure 3 panel A and panel B showing income support over time. For each cohort, if we extrapolate their pre reform trend in DI exits and compare those extrapolations with the actual DI exits in 2015 and 2016, it becomes clear that relative to 2014, less than 2% of individuals had been removed due to the reform by the end of 2015.^29^ And looking at unemployment benefits we see that less than 3% had been moved onto unemployment benefits by the end of 2016. Therefore, it is unlikely that the effect observed for nervous system scripts is due to the loss of income of so few. Further, Figure A3 shows the difference in the average number of scripts for the nervous system for two specific younger cohorts compared to the older cohorts over time namely (i) those who were on DI in all calendar years from 2011 to 2016 (and were not on unemployment benefits) and (ii) those who were not on DI in at least one calendar year between 2014 and 2016. It is noteworthy that even those who stayed on DI had significantly higher nervous system scripts after the reform relative to those not targeted by the reform. This suggests that over and beyond benefit loss, the reform was stressful even for those who were always eligible. This is consistent with the finding in the previous section that those aged 34 at the time of the reform had increased nervous system scripts in 2014 relative to the untreated group although their likelihood to be on unemployment benefits had not increased yet relative to that group. Those who left DI had about the same amount of scripts in 2012 and their scripts became lower over time including after 2014. Overall, the difference between the Young and Old group is positive for those who stayed on DI and negative for those who left suggesting that our overall effect is unlikely to be driven by those who left. It is important to note that this sample split is made on post treatment characteristics and therefore is not exogenous but the results still provide a useful descriptive analysis.

### 5.5 Pre‐reform placebos

To understand whether the results of our DD regression are indeed due to the July 2014 reform, we performed several tests. We removed the years after the reform (2015 and 2016) and tested whether the interaction term between being in the treatment group and observed in years after our placebo reform was significant (see Appendix Table A2). Using 2011 (with post treatment years being 2012–2014), 2012 (with post treatment years being 2013 and 2014) and 2013 (with only 2014 as the post treatment year) as placebo reform years, the interaction term for each placebo reform was small and never significant. Only the interaction term for the nervous system scripts with 2013 as placebo reform year was significant at 10% and with the opposite sign of what we find in our double difference regressions. These tests strengthen confidence that the increased healthcare use we observe for the treated group is indeed due to the reform.

### 5.6 What are the costs of the reform?

Our analysis has shown how many additional scripts and visits were incurred by the reform. We use the 2016 average costs for the treated group (Table A3 in the Appendix reports these averages) to shed light on the costs associated with the additional healthcare use. With an average subsidy per script of $60, the total government spending is equivalent to 349,017 AUD^30^ over 2015 and 2016. For individuals, as most scripts for the nervous system are heavily subsidized, the out of pocket costs are at 44,353 AUD given the average out of pocket per script of 7.54 AUD.

As we demonstrated in the previous section, the reform also led to increase GP and Specialist visits. With an average out of pocket costs at 1.31 AUD for GP and 23.69 AUD for Specialist, the total out of pocket costs are 13,751 AUD for GP and 62,294 AUD for Specialist. For the government, the average subsidy for a GP visit is 50.00 and 107.90 for Specialist which amount to 525,832 AUD and 283,680 AUD for our treated group over the years 2015 and 2016.

In total, the reform led to more than 1.2 million additional Australian dollars spent by the government on subsiding healthcare use for the targeted group. Including the out of pocket expenditures for those targeted by the reform 1.35 million AUD were spent on extra healthcare (1 million USD). Our estimates solely reflect part of the healthcare system cost. The additional visits also incur transaction costs (such as carer time) which may be particularly high for people with disabilities.

These estimations are solely based on the number of individuals included in our regressions. As explained in the method section, we restricted “those under 35” to be 29–31 years old. Assuming a similar increase in scripts for those aged 22–34 years old, we find that the reform incurred an additional 1.6 million in subsidy and slightly over 200,000 AUD in out of pocket expenditure. Repeating this exercise for GP visits, we find that 1.8 million AUD were spent on subsidy and over 50,000AUD in out of pocket. Finally, Specialist visits with an average out of pocket cost at 23 AUD and subsidy at 107 AUD led to 950,000 additional AUD spent on subsidy and 220,000 AUD on out of pocket expenditures.

It is difficult to estimate exactly how much reduction in welfare payments were linked to the reform. Given that people were moved to unemployment benefits, we can look at the difference between unemployment and DI benefits. A single individual with no children would receive 794.8AUD per fortnight if on DI in 2016 while the equivalent would be 527.6 if on unemployment benefits. With a 2% point entry rate into unemployment benefits, the average savings for all those aged 22–34 years old would be at about 25 million AUD. These estimations assume that those who exit DI for unemployment benefits do not re‐enter DI until the end of 2016. These estimations are also solely based on the 2015 and 2016 years. Savings would continue if people remained off DI.

From the government perspective, the savings are at about 25 million AUD. The costs in healthcare subsidies are at 4.3 million AUD in healthcare subsidies. Those costs comes on top of the 21 million Australian dollars allocated for the financial years 2015 and 2016 by the Department of Human Services to conduct those reviews (Australian National Audit Office, 2016).^31^ Therefore, the savings seem to be at about 0.5 million AUD. Among those who were removed half appealed the decision which is likely to incur a significant cost to the judicial system and that we cannot account for in this study. So while the reduction in the numbers on DI reduced the welfare payments these were at least partially offset by additional expenditure elsewhere.

## 6 CONCLUSION

This paper has used unique social welfare data linked to healthcare use data to understand the effect of the 2014 reassessment of DI recipients. We find that this policy led to an increase in nervous system scripts (which includes scripts for anti‐depressants). On average, individuals were prescribed 0.13 extra scripts per year after the reform. This translates into approximately 6000 number of additional scripts for 2015 and 2016. There is no such increase in other types of scripts suggesting that the effect is likely to be due to stress. Combining the increase in the number of scripts with the increase in the number of GP and Specialist visits in the 2 years after the reform and for the 22,281 individuals in our treated group translates into approximately 1.35 million AUD spent on medical services because of the reform. This estimation is likely to be an underestimate due to the fact that our treated population only includes a subsample of those treated.

Overall, under the assumption that the coefficient on the interaction term represents the causal effect of the reform and the increase in nervous system scripts filled are indicative of increased stress,^32^ the results are suggestive of an increase in stress experienced by those targeted by the 2014 reform. It is worth highlighting that not all individuals experiencing stress will go to see their doctor or will be prescribed anti‐depressants and it is therefore possible that this only reflects a part of the potential consequences of the reform on stress and mental health. Although the reform aimed at encouraging those on DI to look for work, we find that very few were actually removed from DI and most of these ended up staying on welfare payments. Given the very few individuals removed, the high cost to the medical system in addition to the likely cost on the judicial system and the likely stress and mental health impacts that individuals seem to have been put through, it is important to account for such unintended consequences when deciding whether or not or how to implement these reassessments in future policy reforms.

## CONFLICT OF INTEREST STATEMENT

The authors declare no conflicts of interest.

## Supporting information

Supporting Information S1

## Data Availability

The data that support the findings of this study are available from the Australian Bureau of Statistics. Restrictions apply to the availability of these data.

## ENDNOTES

^1^
See Name Withheld, *Submission 16*, pp. 4, 6; Name Withheld*, Submission 5*, p. 4. Cited in Joint Committee of Public Accounts and Audit (2017) or (Collie, 2015).

^2^
Prices and exchange rate (using OECD's PPP for GDP) for 2016 used for all costs and conversions. Available at: https://stats.oecd.org/index.aspx?DataSetCode=SNA_Table4 (accessed on 22 March 2022).

^3^
The tables can be found in the *Social Security (Tables for the Assessment of Work‐related Impairment for Disability Support Pension) Determination 2011* which can be accessed through the Comlaw website: http://www.comlaw.gov.au/. A single medical condition may result in multiple functional impairments rated in several Tables while multiple conditions could cause a common impairment under a single Table. Each Table assigns a rating in proportion to the severity of impact of the impairment on function as it relates to work performance. For example, Table 7 is on brain function. A persion with a condition that impacts their neurological or cognitive function so that they cannot remember routines, regular tasks or instructions without being reminded at least everyday would be assigned 20 points in Table 7. The total number of points in each table is then compared to the 20‐point threshold.

^4^
To understand how the Impairment Tables were changed, see the final report of the Advisory Committee to the Australian Government Department of Families, Housing, and Community Serivces and Indigenous Affairs (2011).

^5^
DI can be granted on manifest grounds if the person has a terminal illness (with significantly reduced work capacity during this period), has permanent blindness, has an intellectual disability with an IQ of less than 70, requires nursing home level care, has category 4 HIV/AIDS, or is in receipt of a Department of Veterans' Affairs disability pension at special rate (totally and permanently incapacitated (TPI)).

^6^

https://www.dss.gov.au/sites/default/files/documents/06_2014/fact_sheet_dsp_review_assessment_final.pdf.

^7^
This is the number of cancellations before appeals.

^8^
DI recipients can appeal under Sections 131 and 145 of the *Social Security (Administration) Act 1999*. As at 30 October 2015, 1448 of cancellation were appealed, that is 56.5 per cent of the cancellations (Australian National Audit Office, 2016).

^9^
See Figure A1 in the Online Appendix for removals over time.

^10^
AATA 172:Secretary, Department of Social Services versus Kathryn Krishna ‐ General Division of the Administrative Appeals Tribunal ‐ File Number 2015/3083, 2016, paragraph 19, http://www.austlii.edu.au/cgi‐bin/viewdoc/au/cases/cth/AATA/2016/172.html.

^11^
SA1s are the smallest routine geographic areas available from the Australian Bureau of Statistics and have a population between 200 and 800 people with an average of approximately 400 people (Australian Bureau of Statistics, 2021).

^12^
The majority of scripts are out‐of‐hospitals prescriptions. We exclude out‐of‐hospitals prescriptions that are not subsidized (“under co‐payment”) because there are no data before April 2012. We use out‐of‐hospitals prescriptions that are subsidized (“above co‐payment’’).

^13^
Other medications are included in the nervous system scripts which can also treat mental health problems such as anxiolytics or antipsychotics.

^14^
MBS and PBS provide information on subsidized healthcare use although some unsubsidized drugs can be written on a PBS script because they determine eligibility for safety nets. Services provided to public patients in public hospitals as well as unsubsidized private sector health services are not available in the MBS and PBS data. Most drugs and primary care are however provided through Medicare and therefore will be in the MBS and PBS data. For more on the Australian healthcare system, see (Duckett, 2018).

^15^
The proportion of those on DI in 2011 who actually entered between 2008 and 2011 is examined in the next section. Those with known manifest impairment or limited work capacity were not targeted.

^16^
The Methodology section explains why we have focused on these age groups.

^17^
Exact date of birth is not available and the age given in the MADIP data is as of 9^th^ of August 2016, date of the 2016 Census although age is available for everybody, whether or not they responded to the Census.

^18^
The graphs are similar whether displaying removal numbers or removal rates.

^19^
Table A4 shows that our results are robust to alternative age selection criteria with higher healthcare use for the treated group after 2014 relative to the control group. The point estimates vary which is consistent with aggregating groups that are treated at different times. We present the results for completeness although groups with different treatment patterns should not be aggregated together.

^20^
MADIP BLE data consist of calendar year information (January to December) and the reform took place in July 2014. Therefore, it is not clear whether 2014 should be treated as a pre or post year. Using the DOMINO dataset, we have shown that most individuals were removed during the 2015 and 2016 calendar (Figure 2). Table A4 Panel A shows that removing the year 2014 does not alter the results.

^21^
There is a priori no reason to adjust for individual or local characteristics because the policy was implemented nationally regardless of the characteristics of the participants. The Results section shows that the results are robust to different specifications.

^22^
Table A5 also provides the results of a doughnut regression discontinuity design which is not our preferred strategy due to the large doughnut hole and lack granular data for age resulting in imprecize estimates and lack of clear functional form.

^23^
The appendix reports the value of ${\hat{\beta }}_{5}$ for all regression results including those where the p‐value is above 0.05. Results from equation (3) are reported in Table A7 for the main outcomes.

^24^
This corresponds to the expected average hypothetical difference between the Young group and the Old group in 2015 and 2016 due to their pre reform trends (${\hat{\beta }}_{5}$∗(4 + 5)/2) minus the average difference in the pre reform period (${\hat{\beta }}_{5}$∗(1 + 2 + 3)/4) due to their pre reform trends.

^25^
Historical rates available at https://www.servicesaustralia.gov.au/historical‐versions‐guide‐to‐australian‐government‐payments?context=1#a7 (accessed 20.12.2022).

^26^
An SA2 represents a community that interacts together socially and economically. SA2s have a population between 3000 and 25,000 individuals with of 10,000 people (Australian Bureau of Statistics, 2016).

^27^
Table A4 Panel A in the Appendix presents results with individual fixed effects for all outcomes and the results are similar. Table A5 shows results based on a regression discontinuity design (RDD). We present results for both the RDDs of interest (after the reform took place) and the placebo RDDs (before the reform). We are however cautious in interpreting the results of the RDD due to the large doughnut hole around the age cut off and the lack of granular data for age (i.e., no month of birth available). It is however noteworthy that the coefficient for the RDD is very similar to what we find in our main specification. The placebo RDDs do not find any effect of the reform. Another placebo approach consisted in checking whether there were any particular patterns in nervous system script fillings using an alternative sample of individuals on unemployment benefits in 2011 (and not on DI in 2011). Figure A2 shows the nervous system script fillings over time of different age cohorts for this placebo sample. Unlike the graphs for those on DI in 2011 (Figure 3, panel A), Figure A2 does not display distinct patterns for cohorts age under 35 compared to older cohorts.

^28^
Table A1 presents descriptive statistics for the scripts. Table A9 reports the estimations for all coefficients in Equation (1); Table A10 reports the estimations when the pre trend coefficients are included (Equation 3).

^29^
Figure A4 in the Appendix is based on Roth (2022)'s study and helps visualize the extrapolation of DI exit rates for the young treated group relative to the old control group. Table A8 also provides the results from the regression of Equation (1) with DI as an outcome.

^30^
507,850 USD.

^31^
These costs are in addition to the 10 million the government allocated for the compulsory participation requirements of recipients aged less than 35 years old.

^32^
This interpretation is also followed in (Saxby et al., 2020) who also use MADIP data.

## References

[hec4680-bib-0001] Advisory Committee to the Australian Government Department of Families, Housing, & Community Serivces and Indigenous Affairs . (2011). Review of the tables for the assessment of work‐related impairment for disability support pension. Retrieved from: https://www.afdo.org.au/wp‐content/uploads/2021/06/2011‐Review‐of‐Impairment‐Tables‐for‐DSP.pdf

[hec4680-bib-0002] Anderson, M. L. (2008). Multiple inference and gender differences in the effects of early intervention: A reevaluation of the Abecedarian, Perry Preschool, and Early Training Projects. Journal of the American Statistical Association, 103(484), 1481–1495. 10.1198/016214508000000841

[hec4680-bib-0029] Australian Bureau of Statistics . (2021). Statistical Area Level 1. ABS. https://www.abs.gov.au/statistics/standards/australian‐statistical‐geography‐standard‐asgs‐edition‐3/jul2021‐jun2026/main‐structure‐and‐greater‐capital‐city‐statistical‐areas/statistical‐area‐level‐1#cite‐window1

[hec4680-bib-0003] Australian National Audit Office . (2016). Qualifying for the disability support pension. (18 2015‐2016). Retrieved from: https://www.anao.gov.au/sites/default/files/ANAO_Report_2015‐2016_18.pdf

[hec4680-bib-0004] Barr, B. , Taylor‐Robinson, D. , Stuckler, D. , Loopstra, R. , Reeves, A. , & Whitehead, M. (2016). ‘First, do no harm’: Are disability assessments associated with adverse trends in mental health? A longitudinal ecological study. Journal of Epidemiology & Community Health, 70(4), 339–345. 10.1136/jech-2015-206209 26573235 PMC4819657

[hec4680-bib-0005] Bound, J. (1989). The health and earnings of rejected disability insurance applicants. The American Economic Review, 79(3), 482–503. 11616493

[hec4680-bib-0006] Broadway, B. , Chigavazira, A. , & Kassenboehmer, S. (2014). Labour force potential of disability support pension recipients. In Melbourne: Melbourne institute of applied economic and social research.

[hec4680-bib-0007] Chen, S. , & Van der Klaauw, W. (2008). The work disincentive effects of the disability insurance program in the 1990s. Journal of Econometrics, 142(2), 757–784. 10.1016/j.jeconom.2007.05.016

[hec4680-bib-0008] Collie, A. (2015). Extra medical tests for disability support can make health worse. The Conversation. Retrieved from: https://theconversation.com/extra‐medical‐tests‐for‐disability‐support‐can‐make‐health‐worse‐36984

[hec4680-bib-0009] Curnock, E. , Leyland, A. H. , & Popham, F. (2016). The impact on health of employment and welfare transitions for those receiving out‐of‐work disability benefits in the UK. Social Science and Medicine, 162, 1–10. 10.1016/j.socscimed.2016.05.042 27318626 PMC4962812

[hec4680-bib-0010] David, H. , Duggan, M. , Greenberg, K. , & Lyle, D. S. (2016). The impact of disability benefits on labor supply: Evidence from the VA's disability compensation program. American Economic Journal: Applied Economics, 8(3), 31–68. 10.1257/app.20150158

[hec4680-bib-0011] Department of Social Services ‐ Australian Government . (2016). Disability support pension revised impairment tables ‐ post implementation review.

[hec4680-bib-0012] Deshpande, M. (2016). Does welfare inhibit success? The long‐term effects of removing low‐income youth from the disability rolls. The American Economic Review, 106 11, 3300–3330. 10.1257/aer.20151129 29552877

[hec4680-bib-0013] Duckett, S. (2018). Expanding the breadth of Medicare: Learning from Australia. Health Economics, Policy and Law, 13(3–4), 344–368. 10.1017/S1744133117000421 29362017

[hec4680-bib-0014] French, E. , & Song, J. (2014). The effect of disability insurance receipt on labor supply. American Economic Journal: Economic Policy, 6(2), 291–337. 10.1257/pol.6.2.291

[hec4680-bib-0015] Garcia‐Gomez, P. , & Gielen, A. C. (2018). Mortality effects of containing moral hazard: Evidence from disability insurance reform. Health Economics, 27(3), 606–621. 10.1002/hec.3617 29237234

[hec4680-bib-0016] Gelber, A. , Moore, T. , & Strand, A. (2018). Disability insurance income saves lives. Unpublished paper.

[hec4680-bib-0017] Henriques‐Gomes, L. (2020). Sick or disabled people on Newstart face 'unrealistic' obligations to find work. The Guardian. Retrieved from: https://www.theguardian.com/australia‐news/2020/mar/05/sick‐or‐disabled‐people‐on‐newstart‐face‐unrealistic‐obligations‐to‐find‐work?CMP=share_btn_tw

[hec4680-bib-0018] Joint Committee of Public Accounts and Audit . (2017). Commonwealth risk management: inquiry based on Auditor‐General's report 18 (2015‐16) (9781743666081 (print)). Parliament of the Commonwealth of Australia. Retrieved from: http://www.aph.gov.au/Parliamentary_Business/Committees/Joint/Public_Accounts_and_Audit/CRM/Report_461

[hec4680-bib-0019] Jones, M. K. (2008). Disability and the labour market: A review of the empirical evidence. Journal of Economics Studies, 35(5), 405–424. 10.1108/01443580810903554

[hec4680-bib-0020] Kendrick, T. (2021). Strategies to reduce use of antidepressants. British Journal of Clinical Pharmacology, 87(1), 23–33. 10.1111/bcp.14475 32656861

[hec4680-bib-0021] Koning, P. , & Lindeboom, M. (2015). The rise and fall of disability insurance enrollment in The Netherlands. The Journal of Economic Perspectives, 29(2), 151–172. 10.1257/jep.29.2.151 28443648

[hec4680-bib-0022] Low, H. , & Pistaferri, L. (2020). Disability insurance: Theoretical trade‐offs and empirical evidence. Fiscal Studies, 41(1), 129–164. 10.1111/1475-5890.12215

[hec4680-bib-0023] Moore, T. J. (2015). The employment effects of terminating disability benefits. Journal of Public Economics, 124, 30–43. 10.1016/j.jpubeco.2015.02.004

[hec4680-bib-0024] Parliamentary Budget Office . (2018). Disability support pension. Historical and projected trends. Parliament of Australia. (01/2018).

[hec4680-bib-0025] Roth, J. (2022). Pretest with caution: Event‐study estimates after testing for parallel trends. The American Economic Review: Insights, 4(3), 305–322. 10.1257/aeri.20210236

[hec4680-bib-0026] Saxby, K. , de New, S. C. , & Petrie, D. (2020). Structural stigma and sexual orientation disparities in healthcare use: Evidence from Australian Census‐linked‐administrative data. Social Science and Medicine, 255, 113027. 10.1016/j.socscimed.2020.113027 32408084

[hec4680-bib-0027] Von Wachter, T. , Song, J. , & Manchester, J. (2011). Trends in employment and earnings of allowed and rejected applicants to the social security disability insurance program. The American Economic Review, 101(7), 3308–3329. 10.1257/aer.101.7.3308 PMC5648004 29056750

