# Supplementary material for: The impact of Disability Insurance reassessment on healthcare use
Source: Health Econ. 2023 Apr 1;32(7):1581–602. doi: 10.1002/hec.4680 (PMC10947330; doi:10.1002/hec.4680)
Supplement: Supplementary file 1 — Supporting Information S1 [file HEC-32-1581-s001.docx]

**Online appendix**

**Figure A1** Number of removals from DI after January 2014 by semester for each one-year age group because of having less than 20 impairment points or having a short term disability


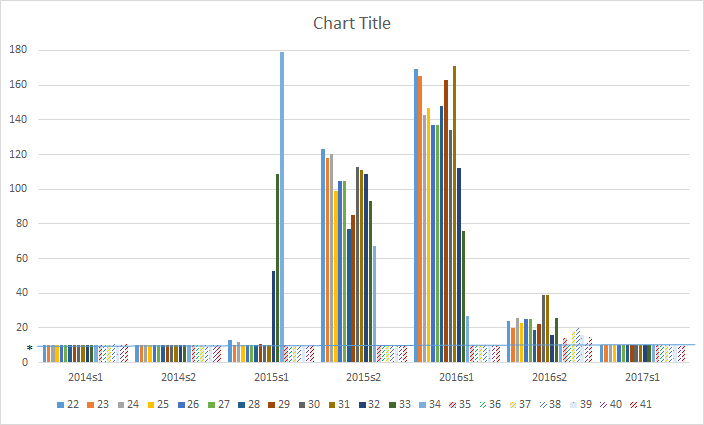


*Source:* Authors’ own calculation from the Australian Department of Social Services Data Over Multiple Individual Occurrences (DOMINO) dataset

*Notes:* Age is defined on 01.07.2014, date of the 2014 reform. Numbers below 10 are bottom-coded at 10 to ensure confidentiality. The figures look similar whether exit numbers or exit rates are shown.

The hatched aged groups are those 35 and above who were not targeted by the reform. As can be seen they are indeed unlikely to be removed whether before or after the reform. Other age groups are mainly affected in the financial year 2015-2016. Nobody seems to be affected in 2014 although the reform occurred on 01.07.2014. This might be because the review process takes time (first a letter is received, then a medical examination takes place) before someone is actually removed. It is noteworthy that only a few age groups (vertical bars) were targeted in the first half of 2015 compared with the second half of 2015 or the first half of 2016.

**Figure A2:** Nervous system scripts for those on unemployment benefits in 2011 but not on DI in 2011

*Source:* MADIP Basic Longitudinal Extract 2016 data.

*Notes:* Age cohorts are defined based on their age as of 09.08.2014. Our sample consists of individuals linked to the Medicare Enrollments Database (MEDB) and aged 29 to 38 years old in 2014 who received at least one payment from DI in 2011 and who had any medical visit or medical service used, prescription, tax form filled, social security payment or who responded to the census in 2016. We show separately those who were 32, 33, 34 and 35 years old as they are not part of our main sample.

**Figure A3:** Nervous system scripts for those who were on DI until the end of 2016 and those who left DI after the reform

**
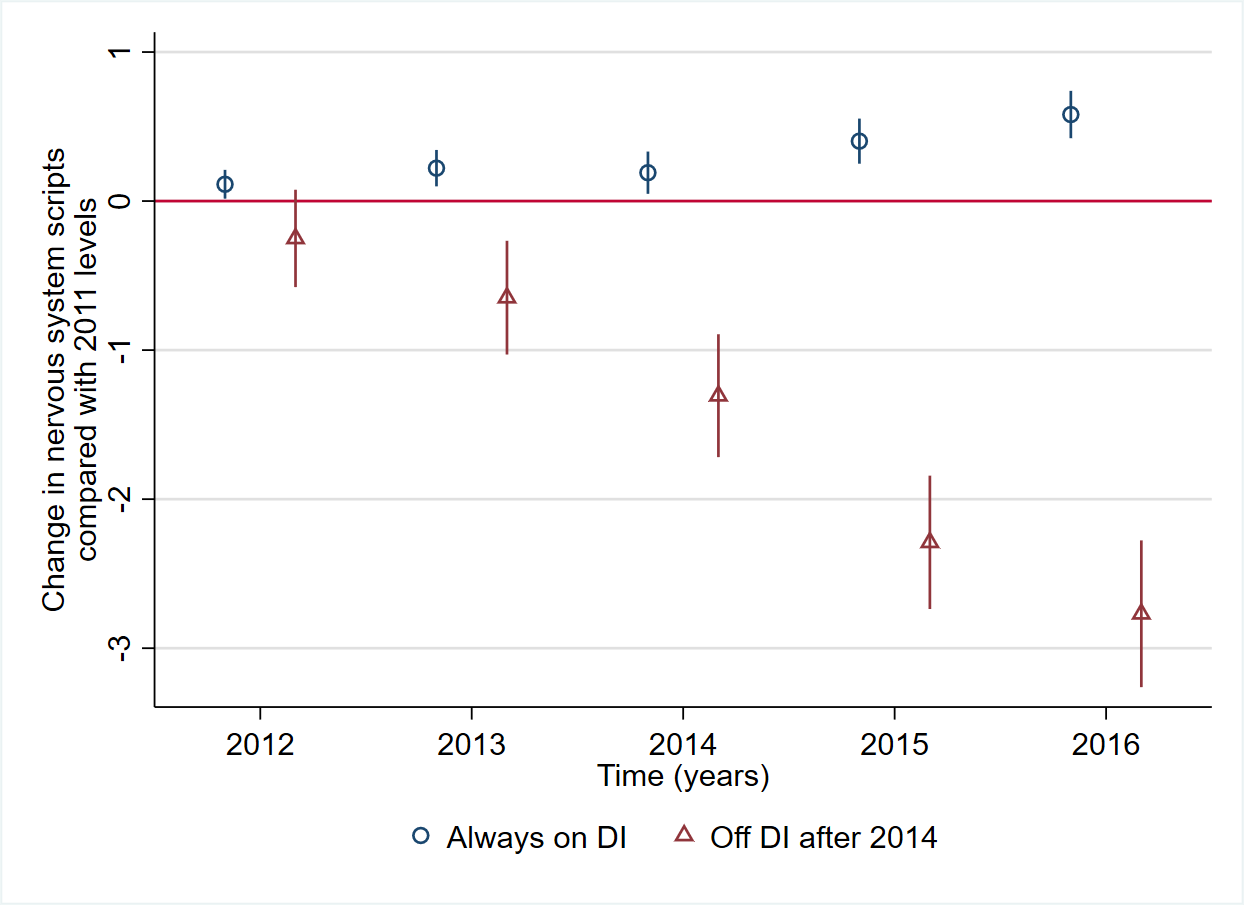
**

*Source:* MADIP Basic Longitudinal Extract 2016 data.

*Notes:* We keep the same control group but now compare this group with two subgroups of our young treated population – those always on DI and those who left DI after 2014 (defined below).

**Always on DI** – DI from 2011-2016 and no unemployment benefits from 2012 till 2016

**Off DI after reform**– Of those where there was no evidence they had left DI before the end of 2014 (on DI from 2011 till at least sometime in 2014 and no unemployment benefits from 2012 till 2014) we select those who either were (A) NOT on DI in 2015 or 2016 OR (B) were on DI at some point in 2015 and 2016 but who also were on unemployment benefits at some point in 2015 or 2016.

**Figure A4:** Probability to be on DI benefits for those in the treated group relative to the control group.

The figure below helps understand whether and to what extent the reform led to DI exits. The coefficients (black circles) are the result from an event study regression. They correspond to the probability to be on DI for the young group relative to the old group. The red line with red triangles corresponds to the hypothesized trend which is based on the linear difference between the young and old group before the reform. This difference is extended beyond 2014 to understand whether our estimated coefficient for the post treatment years are the result of differences in trends between the young and old group before the reform. Clearly the last coefficient on the right, corresponding to the year 2016 is well below the hypothesized trends suggesting that the difference in the probability to be on DI between the two groups is very unlikely to be due to differences in trends before the reform. This suggests that the reform led to a significantly lower probability though a small effect to be on DI in 2016 for the young group which means that they exited as early as 2015.


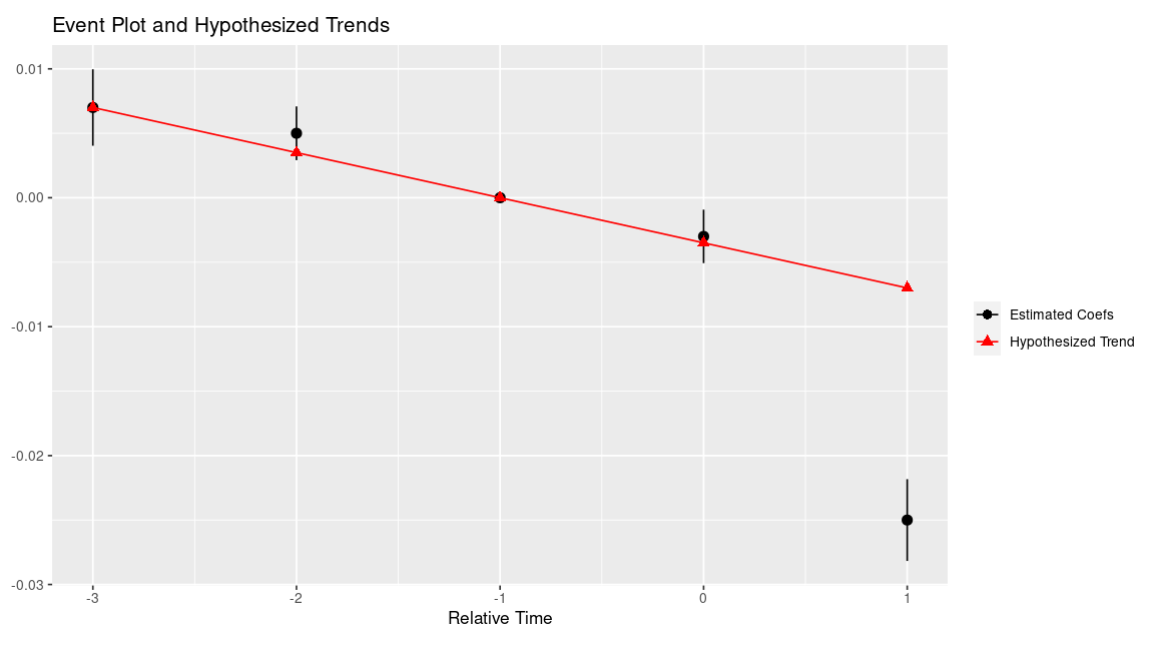


*Source:* Roth (2022) *pretrends* package used to make the figure based on MADIP Basic Longitudinal Extract 2016 data.

*Notes:* The estimated coefficients (black dots) correspond to the probability to be on DI for the young group relative to the old group. The first treated year, 2015 is on the 0 point. The hypothesized trend in red corresponds to the linear difference between the young and old group before the reform and is extended beyond 2014.

**Table A1:** Mean and standard deviations for the scripts in the treated and control groups in 2011 and 2016 (complements Table 2).

|  | 2011 | | 2016 | |
| --- | --- | --- | --- | --- |
|  | Treated Group  Younger  (29-31 y.o.) | Control Group  Older  (36-38 y.o.) | Treated Group  Younger  (29-31 y.o.) | Control Group  Older  (36-38 y.o.) |
|  |  |  |  |  |
| Alimentary System scripts | 1.555 (4.361) | 2.079 (5.052) | 2.204 (5.376) | 2.954 (6.257) |
|  |  |  |  |  |
| Cardiovascular Scripts | 0.755 (3.306) | 1.453 (4.741) | 1.263 (4.331) | 2.330 (5.988) |
|  |  |  |  |  |
| Respiratory System Scripts | 0.724 (2.942) | 0.900 (3.343) | 0.834 (3.219) | 1.072 (3.756) |
|  |  |  |  |  |
| Blood, blood forming organs | 0.160 (1.219) | 0.243 (1.561) | 0.209 (1.437) | 0.310 (1.776) |
|  |  |  |  |  |
| Anti-infectives | 1.770 (3.148) | 1.857 (3.251) | 1.671 (3.186) | 1.887 (3.548) |
|  |  |  |  |  |
| Sensory organs | 0.256 (1.511) | 0.285 (1.576) | 0.259 (1.721) | 0.284 (1.875) |
|  |  |  |  |  |
| Systemic hormonal | 0.284 (1.587) | 0.318 (1.537) | 0.338 (1.640) | 0.378 (1.641) |
|  |  |  |  |  |
| Antineoplastic and immunomodulating agents | 0.144 (1.289) | 0.189 (1.466) | 0.279 (2.030) | 0.328 (2.188) |
|  |  |  |  |  |
| Antiparasitic, insecticides | 0.0103 (0.149) | 0.0124 (0.167) | 0.0109 (0.163) | 0.0134 (0.218) |
|  |  |  |  |  |
| Various scripts | 0.0216 (0.448) | 0.0236 (0.378) | 0.0246 (0.544) | 0.0313 (0.482) |
|  |  |  |  |  |
| Other scripts | 0.00812 (0.186) | 0.0105 (0.184) | 0.00925 (0.217) | 0.0117 (0.275) |
| Observations | 22,281 | 27,321 | 22,281 | 27,321 |

*Source:* MADIP Basic Longitudinal Extract 2016 data.

*Notes:* Our sample consists of individuals linked to the Medicare Enrollments Database (MEDB) and aged 29 to 38 years old in 2014 who received at least one payment from DI in 2011 and who had any medical visit or medical service used, prescription, tax form filled, social security payment or who responded to the census in 2016. We excluded those who were 32, 33, 34 and 35 to match the sample used in the regressions. The number of prescriptions is the total for a calendar year.

**Table A2**: Difference-in-difference regression on healthcare use outcomes for placebo policy occurring before 2014 (years 2015 and 2016 removed).

|  | Scripts | Medical visits | |
| --- | --- | --- | --- |
|  | Nervous system | GP | Specialist |
| Young*Post2011 | -0.010 | 0.030 | 0.008 |
|  | (0.052) | (0.047) | (0.026) |
| Young*Post2012 | -0.039 | 0.031 | 0.006 |
|  | (0.050) | (0.044) | (0.024) |
| Young*Post2013 | -0.096* | 0.036 | 0.016 |
|  | (0.053) | (0.047) | (0.026) |
| Observations | 198,408 | 198,408 | 198,408 |

*Source:* MADIP Basic Longitudinal Extract 2016 data.

*Notes:* * p<.1, ** p<.05, *** p<.01; standard errors in parentheses clustered at the individual level. Each cell corresponds to the results from a separate differences-in-differences regression.

**Table A3:** Average use and costs associated with the key MBS and PBS items for the young/treated group in 2016

| PBS item | Number of scripts | Benefits  received | Patient  contribution | Out of  pocket | Average  subsidy | | Total: out of pocket + subsidy |  |
| --- | --- | --- | --- | --- | --- | --- | --- | --- |
|  |  |  |  |  |  | |  |  |
| Nervous | 9.50 | 563.5 | 71.61 | 7.54 | 59.33 | | 44,353+349,017 |  |
| system | (11.20) | (1,134.5) | (110.0) |  |  | | =393,370 |  |
|  |  |  |  |  |  | |  |  |
|  |  |  |  |  |  | |  |  |
| MBS items | Number of visits | Benefits  paid | Fees  charged | Out of  pocket | Average  subsidy | | Total: out of pocket + subsidy |  |
|  |  |  |  |  |  | |  |  |
| GP visits | 7.65 | 382.4 | 392.4 | 1.31 | 50.00 | | 13,751+525,832 |  |
|  | (7.64) | (485.8) | (494.8) |  |  | | =539,582 |  |
|  |  |  |  |  |  | |  |  |
| Specialist | 1.72 | 185.8 | 226.6 | 23.69 | 107.90 | | 62,294+283,680 |  |
| visits | (4.23) | (546.0) | (629.2) |  |  | | =345,974 |  |
|  |  | | | | |  | | |
| Total costs | AUD | | | | | 1,354,971  (120,398+1,234,573) | | |

*Source:* Authors’ own calculations from MADIP Basic Longitudinal Extract 2016 data.

**Table A4:** Regressions of healthcare use outcomes and welfare recipiency for alternative specifications and alternative samples

| Interaction term  Young*Post2014 | Nervous system  scripts | GP visits | Specialist visits |
| --- | --- | --- | --- |
| Panel A: Main sample: 29-31 vs 36-38 | | |  |
| Diff in Diff | 0.132** | 0.236*** | 0.059** |
|  | (0.053) | (0.044) | (0.024) |
| Individual FE | 0.132** | 0.236*** | 0.059** |
|  | (0.053) | (0.044) | (0.024) |
| Diff in Diff | 0.154*** | 0.242*** | 0.079*** |
| excluding 2014 | (0.057) | (0.046) | (0.025) |
| Panel B: Varying the age of the treated/young group (Diff in Diff) | | | |
| 34 vs 36-38 | *0.230^^^* | 0.012 | -0.032 |
|  | *(0.077)* | (0.064) | (0.033) |
| 33 vs 36-38 | 0.120 | 0.071 | -0.006 |
|  | (0.076) | (0.064) | (0.034) |
| 32 vs 36-38 | 0.261*** | 0.214*** | *0.102^^^* |
|  | (0.077) | (0.063) | *(0.035)* |
| 29-32 vs 36-38 | 0.165*** | 0.230*** | 0.070*** |
|  | (0.049) | (0.041) | (0.022) |
| 29-33 vs 36-38 | 0.156*** | 0.196*** | 0.054** |
|  | (0.047) | (0.040) | (0.021) |
| 29-34 vs 36-38 | 0.169*** | 0.164*** | 0.039* |
|  | (0.045) | (0.038) | (0.020) |
| 26-31 vs 36-38 | *0.102^^* | 0.280*** | 0.034* |
|  | *(0.045)* | (0.038) | (0.020) |
| 23-31 vs 36-38 | *0.095^^* | 0.300*** | 0.024 |
|  | *(0.042)* | (0.036) | (0.019) |
| Panel C: Different age for the treated and control groups (Diff in Diff) | | | |
| 29-32 vs 36-39 | 0.196*** | 0.260*** | 0.076*** |
|  | (0.046) | (0.038) | (0.021) |
| 29-31 vs 36-39 | 0.163*** | 0.265*** | 0.065*** |
|  | (0.049) | (0.041) | (0.022) |
| 29-31 vs 36-40 | 0.153*** | 0.262*** | 0.069*** |
|  | (0.047) | (0.039) | (0.021) |
| 28-32 vs 36-40 | 0.167*** | 0.265*** | 0.067*** |
|  | (0.040) | (0.034) | (0.018) |
| 28-31 vs 36-39 | 0.146*** | 0.275*** | 0.051** |
|  | (0.045) | (0.038) | (0.021) |

*Source:* MADIP Basic Longitudinal Extract 2016 data.

*Notes:* * p<.1, ** p<.05, *** p<.01; ^ p<.1, ^^ p<.05, ^^^ p<.01 for cells in italics; standard errors in parentheses clustered at the individual level. Cells in italics are those for which the probability that the pre 2014 trends between the young and old group is the same is below 10%. Full table of regression results available upon request. Results from the difference in difference with pre reform trend and pre reform trend interacted with the young group dummy also available upon request.

**Table A5:** Regression Discontinuity Design on scripts for the nervous system

|  | 2011 | 2012 | 2013 | 2014 | 2015 | 2016 |
| --- | --- | --- | --- | --- | --- | --- |
| Young (Estimated Treatment Effect) | **0.164** | **0.158** | **0.051** | **0.038** | **0.224** | **0.432*** |
|  | **(0.220)** | **(0.223)** | **(0.226)** | **(0.233)** | **(0.235)** | **(0.236)** |
| x_c | -0.113*** | -0.102*** | -0.075*** | -0.069** | -0.072** | -0.093*** |
|  | (0.027) | (0.027) | (0.027) | (0.028) | (0.028) | (0.028) |
| Young # x_c | -0.360*** | -0.373*** | -0.393*** | -0.413*** | -0.419*** | -0.404*** |
|  | (0.039) | (0.039) | (0.04) | (0.041) | (0.041) | (0.041) |
| Constant | 10.443*** | 10.713*** | 10.994*** | 11.540*** | 11.643*** | 11.506*** |
|  | (0.108) | (0.109) | (0.111) | (0.113) | (0.114) | (0.113) |
| Observations | 102,859 | 102,859 | 102,859 | 102,859 | 102,859 | 102,859 |

*Source:* MADIP Basic Longitudinal Extract 2016 data.

*Notes:* * p<.1, ** p<.05, *** p<.01; standard errors in parentheses clustered at the individual level.

The running variable x_c is equal to 35 minus the age as of 01.07.2014.

To estimate the Regression Discontinuity Design, we take in a large sample of the population, namely those aged 26 to 31 and those aged 36 to 41 in 2014. Specifications with a polynomial of order 2 showed that the interaction term with the second order was never significant and therefore a standard linear model is used. For the post treatment years (2015 and 2016), we can see large effects of the reform and even some significant effects in 2016. Other years can be interpreted as placebos and do not show any impact. However, the estimates are very imprecise. It is noteworthy that the effect for the double difference is within the 95% confidence interval of the RDD whether we take the 2015 or 2016 results.

**Table A6:** Difference-in-difference regression on the use of any scripts or visits (extensive margin)

|  | (1) | (2) | | | | (3) | |  |
| --- | --- | --- | --- | --- | --- | --- | --- | --- |
|  | Nervous system scripts (0/1) | GP visits  (0/1) | | | | Specialist visits  (0/1) | |  |
| Young*Post2014 | 0.006** | | 0.012*** | | 0.006* | |  |  |
|  | (0.003) | | (0.002) | | (0.003) | |  |  |
| Young | -0.090*** | | -0.023*** | | -0.015*** | |  |  |
|  | (0.004) | | (0.002) | | (0.003) | |  |  |
| Post2014 | -0.002 | | 0.002 | | 0.012*** | |  |  |
|  | (0.002) | | (0.001) | | (0.002) | |  |  |
| Constant | 0.707*** | | 0.895*** | | 0.353*** | |  |  |
|  | (0.002) | | (0.001) | | (0.002) | |  |  |
| Observations | 297,612 | | 297,612 | 297,612 | | | | |

*Source:* MADIP Basic Longitudinal Extract 2016 data.

*Notes:* * p<.1, ** p<.05, *** p<.01; standard errors in parentheses clustered at the individual level

**Table A7:** Difference-in-difference regression on healthcare use with pre reform trends.

|  | (1) | (2) | (3) |
| --- | --- | --- | --- |
|  | nervous system scripts | GP visits | Specialist visits |
| Constant | 10.282*** | 8.065*** | 1.751*** |
|  | (0.074) | (0.054) | (0.030) |
| Young*Post2014 | 0.073 | 0.276*** | 0.072* |
|  | (0.095) | (0.076) | (0.042) |
| Young | -2.354*** | -1.365*** | -0.094** |
|  | (0.107) | (0.077) | (0.045) |
| Post2014 | 1.480*** | 0.664*** | -0.018 |
|  | (0.066) | (0.053) | (0.028) |
| pre 2014 trend | 0.332*** | 0.140*** | -0.015** |
|  | (0.016) | (0.014) | (0.007) |
| pre 2014 trend * Young | -0.024 | 0.016 | 0.005 |
|  | (0.024) | (0.020) | (0.011) |
| Observations | 297,612 | 297,612 | 297,612 |

*Source:* MADIP Basic Longitudinal Extract 2016 data.

*Notes:* * p<.1, ** p<.05, *** p<.01; standard errors in parentheses clustered at the individual level.

**Table A8:** Main specification results using welfare benefits as outcomes

|  | Welfare Benefits | |  |  |
| --- | --- | --- | --- | --- |
|  | Unemployment | DI |  |  |
| Young*Post2014 | 0.020*** | -0.019*** |  |  |
|  | (0.001) | (0.001) |  |  |
| Young (“treated”) | -0.000 | -0.005*** |  |  |
|  | (0.001) | (0.001) |  |  |
| Post 2014 | -0.008*** | -0.028*** |  |  |
|  | (0.001) | (0.001) |  |  |
| Constant | 0.018*** | 0.980*** |  |  |
|  | (0.000) | (0.001) |  |  |
| Observations | 297,612 | 297,612 |  |  |
| Pretrend diff. (p-val) | 0.600 | 0.000 |  |  |

*Source:* MADIP Basic Longitudinal Extract 2016 data.

*Notes:* * p<.1, ** p<.05, *** p<.01; standard errors in parentheses clustered at the individual level. The last row indicates the probability that the trends were similar for the old and the young groups prior to 2014 (it tests whether the trend before 2014 for the young group was statistically significant conditional on the general trend before 2014 and the covariates of the regression). The group of young includes those aged 29 to 31 years old in 2014. The group of old (control group) includes individuals aged 36 to 38 years old in 2014. In the regressions that include pretrends, the coefficient on the interaction term between the treated dummy and the pretrend is -0.003 for the outcome “DI”.

**Table A9:** Difference-in-difference regression on all scripts

|  | Alimentary System | nervous system | Cardio- vascular | Respiratory System | Blood, blood forming organs | Genitourinary, sex hormones | Anti-infectives | Musculo-skeletal system |
| --- | --- | --- | --- | --- | --- | --- | --- | --- |
| Young*Post2014 | -0.124*** | 0.132** | -0.214*** | -0.027 | -0.013 | 0.000 | -0.046** | 0.026** |
|  | (0.028) | (0.053) | (0.025) | (0.017) | (0.010) | (0.009) | (0.020) | (0.013) |
|  | [0.000] | [0.012] | [0.000] | [0.104] | [0.167] | [0.992] | [0.022] | [0.046] |
|  | {0.001} | {0.055} | {0.001} | {0.210} | {0.314} | {1.000} | {0.071} | {0.113} |
| Young | -0.599*** | -2.414*** | -0.832*** | -0.200*** | -0.097*** | 0.047*** | -0.141*** | -0.223*** |
|  | (0.043) | (0.093) | (0.037) | (0.027) | (0.012) | (0.013) | (0.024) | (0.018) |
| Post 2014 | 0.526*** | 0.650*** | 0.531*** | 0.102*** | 0.053*** | -0.000 | 0.020 | 0.044*** |
|  | (0.020) | (0.036) | (0.019) | (0.012) | (0.007) | (0.006) | (0.014) | (0.010) |
| Constant | 2.374*** | 11.112*** | 1.727*** | 0.952*** | 0.286*** | 0.432*** | 1.850*** | 0.650*** |
|  | (0.031) | (0.065) | (0.029) | (0.019) | (0.009) | (0.009) | (0.017) | (0.013) |
| Observations | 297,612 | 297,612 | 297,612 | 297,612 | 297,612 | 297,612 | 297,612 | 297,612 |
| Pretrend diff. (p-val) | 0.000 | 0.312 | 0.000 | 0.151 | 0.035 | 0.398 | 0.004 | 0.456 |
| Coefficient with correction for pre trends | 0.020 |  | 0.047 |  | 0.014 |  | 0.038 |  |

|  | Sensory organs | Derma-  tology | Systemic hormonal | Antineoplastic and immunomodulating agents | Antiparasitic, insecticides | Various scripts | Other scripts |
| --- | --- | --- | --- | --- | --- | --- | --- |
| Young*Post2014 | -0.002 | 0.005 | 0.004 | 0.005 | -0.000 | -0.002 | -0.000 |
|  | (0.010) | (0.007) | (0.008) | (0.011) | (0.001) | (0.004) | (0.002) |
|  | [0.831] | [0.489] | [0.635] | [0.658] | [0.880] | [0.584] | [0.861] |
|  | {1.000} | {0.918} | {0.918} | {0.918} | {1.000} | {0.918} | {1.000} |
| Young | -0.029** | -0.033*** | -0.043*** | -0.053*** | -0.002** | -0.008** | -0.003* |
|  | (0.013) | (0.007) | (0.013) | (0.013) | (0.001) | (0.004) | (0.002) |
| Post 2014 | 0.014** | 0.001 | 0.034*** | 0.074*** | -0.001 | 0.006** | 0.000 |
|  | (0.007) | (0.005) | (0.006) | (0.007) | (0.001) | (0.003) | (0.001) |
| Constant | 0.295*** | 0.254*** | 0.343*** | 0.241*** | 0.015*** | 0.031*** | 0.011*** |
|  | (0.009) | (0.005) | (0.009) | (0.010) | (0.001) | (0.002) | (0.001) |
| Observations | 297,612 | 297,612 | 297,612 | 297,612 | 297,612 | 297,612 | 297,612 |
| Pretrend diff. (p-val) | 0.986 | 0.532 | 0.130 | 0.190 | 0.572 | 0.001 | 0.860 |
| Coefficient corrected for pre trends |  |  |  |  |  | 0.013 |  |

*Source:* MADIP Basic Longitudinal Extract 2016 data.

*Notes:* * p<.1, ** p<.05, *** p<.01; standard errors in parentheses clustered at the individual level. P values in squared brackets. Sharpened False Discovery Rate (FDR) q-values shown in curly brackets for the interaction term coefficient. The last row indicates the probability that the trends were similar for the old and the young groups prior to 2014 (it tests whether the trend before 2014 for the young group was statistically significant conditional on the general trend before 2014 and the covariates of the regression). For p values below 0.1, we show the coefficient for the interaction term corrected for the young specific trends before the reform, that is we subtract 1.5*the coefficient on the young specific pre trend in the regression of equation (3) shown in table A14. The group of young includes those aged 29 to 31 years old in 2014. The group of old (control group) includes individuals aged 36 to 38 years old in 2014.

**Table A10:** Difference-in-difference regression on all scripts with pre reform trends included as regressors

|  | Alimentary System | Nervous system | Cardio-  vascular | Respiratory System | Blood, blood forming organs | Genitourinary, sex hormones | Anti-infectives | Musculo-skeletal system |
| --- | --- | --- | --- | --- | --- | --- | --- | --- |
| Young*Post2014 | -0.245*** | 0.073 | -0.432*** | -0.053* | -0.037** | -0.008 | -0.117*** | 0.016 |
|  | (0.049) | (0.095) | (0.043) | (0.028) | (0.017) | (0.015) | (0.035) | (0.022) |
| Young(“treated”) | -0.479*** | -2.354*** | -0.613*** | -0.174*** | -0.074*** | 0.056*** | -0.071** | -0.212*** |
|  | (0.046) | (0.107) | (0.040) | (0.031) | (0.015) | (0.016) | (0.034) | (0.021) |
| Post 2014 | 1.016*** | 1.480*** | 0.993*** | 0.178*** | 0.127*** | 0.003 | 0.011 | 0.117*** |
|  | (0.035) | (0.066) | (0.033) | (0.021) | (0.012) | (0.010) | (0.025) | (0.017) |
| pre 2014 trend | 0.196*** | 0.332*** | 0.185*** | 0.031*** | 0.030*** | 0.001 | -0.004 | 0.029*** |
|  | (0.008) | (0.016) | (0.008) | (0.005) | (0.003) | (0.003) | (0.007) | (0.004) |
| pre 2014 trend * | -0.048*** | -0.024 | -0.087*** | -0.011 | -0.009** | -0.003 | -0.028*** | -0.004 |
| Young | (0.012) | (0.024) | (0.010) | (0.007) | (0.004) | (0.004) | (0.010) | (0.006) |
| Constant | 1.884*** | 10.282*** | 1.265*** | 0.876*** | 0.212*** | 0.429*** | 1.860*** | 0.578*** |
|  | (0.033) | (0.074) | (0.031) | (0.023) | (0.011) | (0.010) | (0.023) | (0.016) |
| Observations | 297,612 | 297,612 | 297,612 | 297,612 | 297,612 | 297,612 | 297,612 | 297,612 |

*Source:* MADIP Basic Longitudinal Extract 2016 data.

*Notes:* * p<.1, ** p<.05, *** p<.01; standard errors in parentheses clustered at the individual level. The last row indicates the probability that the trends were similar for the old and the young groups prior to 2014 (it tests whether the trend before 2014 for the young group was statistically significant conditional on the general trend before 2014 and the covariates of the regression). The group of young includes those aged 29 to 31 years old in 2014. The group of old (control group) includes individuals aged 36 to 38 years old in 2014.

|  | Sensory organs | Derma-  tology | Systemic hormonal | Antineoplastic and immunomodulating agents | Antiparasitic, insecticides | Various  scripts | Other  scripts |
| --- | --- | --- | --- | --- | --- | --- | --- |
| Young*Post2014 | -0.002 | -0.001 | -0.010 | -0.010 | -0.001 | -0.014** | -0.001 |
|  | (0.016) | (0.012) | (0.014) | (0.019) | (0.002) | (0.006) | (0.003) |
| Young (“treated”) | -0.029* | -0.027** | -0.029* | -0.038*** | -0.001 | 0.004 | -0.002 |
|  | (0.016) | (0.011) | (0.016) | (0.014) | (0.002) | (0.005) | (0.002) |
| Post 2014 | 0.028** | 0.004 | 0.075*** | 0.160*** | 0.000 | 0.020*** | 0.001 |
|  | (0.011) | (0.008) | (0.010) | (0.013) | (0.002) | (0.004) | (0.002) |
| pre 2014 trend | 0.006** | 0.001 | 0.016*** | 0.034*** | 0.001 | 0.006*** | 0.000 |
|  | (0.003) | (0.002) | (0.003) | (0.003) | (0.001) | (0.001) | (0.001) |
| pre 2014 trend*Young | 0.000 | -0.002 | -0.006 | -0.006 | -0.000 | -0.005*** | -0.000 |
|  | (0.004) | (0.003) | (0.004) | (0.005) | (0.001) | (0.002) | (0.001) |
| Constant | 0.280*** | 0.250*** | 0.302*** | 0.155*** | 0.013*** | 0.018*** | 0.010*** |
|  | (0.011) | (0.008) | (0.010) | (0.010) | (0.001) | (0.003) | (0.001) |
| Observations | 297,612 | 297,612 | 297,612 | 297,612 | 297,612 | 297,612 | 297,612 |
